# An Intervention to Promote Sustainable and Inclusive Eating Behaviors in Primary and Preschools in Italy: A Pilot Study of the SoS-4-SoUPS Project

**DOI:** 10.3390/nu18213700

**Published:** 2026-11-07

**Authors:** Anna Vedani, Maria Jose Habib, Erica DellaBianca, Lucia Rogledi, Nicola Lamberti, Giacomo Fontana, Carola Ricci, Giulia Mattavelli

**Affiliations:** 1 IUSS Cognitive Neuroscience (ICoN) Center, Scuola Universitaria Superiore IUSS, 27100 Pavia, Italy; giulia.mattavelli@iusspavia.it; 2 PlanEat Società Benefit, 20059 Milan, Italy; erica.dellabianca@planeat.eco (E.D.); lucia.rogledi@alimentiamoci.com (L.R.); nicola.lamberti@alimentiamoci.com (N.L.); 3 Cooperativa Rosa Dei Venti, 27049 Pavia, Italy; amministrazione@rosadeiventi.info; 4 Department of Science, Technology and Society, Scuola Universitaria Superiore IUSS, 27100 Pavia, Italy; carola.ricci@unipv.it; 5 Department of Political and Social Sciences, Università Degli Studi Di Pavia, 27100 Pavia, Italy; 6 Cognitive Neuroscience Laboratory of Pavia Institute, Istituti Clinici Scientifici Maugeri IRCCS, 27100 Pavia, Italy

**Keywords:** school-based intervention, food waste, childhood nutrition, digital meal planning, sustainable food systems, dietary behavior, inclusivity, well-being

## Abstract

Background/Objectives: Food waste contributes to environmental, social, economic, and nutritional losses. In this context, school canteens represent a key setting for shaping sustainable and healthy early dietary behaviors. This pilot study aimed to evaluate the implementation of a combined digital meal-planning and educational intervention and to explore changes in plate waste, food-related responses, and perceived inclusivity and involvement. Methods: An uncontrolled pre-post pilot study in one preschool and one primary school in a single municipality was conducted. The intervention combined a digital lunch-planning tool, enabling menu and portion selection, with educational workshops on food and sustainability. Plate waste was weighed during two menu-matched weeks, one before and one after implementation. Parent and child questionnaires assessing dietary habits, emotional responses to food and food waste images, perceived inclusivity and involvement, and well-being were recorded. Results: Of 159 enrolled pupils, 155 registered on the platform and 152 used it at least once. Overall, 82.3% of meals were pre-planned through the platform. Questionnaires were completed at baseline by 93 participants and at follow-up by 60, with 48 participating at both time points. Exploratory analyses identified differences over time in some emotional-response ratings and higher parent-rated inclusivity and involvement at follow-up. Across the two observation weeks, measured plate waste decreased from 0.066 to 0.038 kg per meal attendance, an observed reduction of 42.1%. As portion sizes could be reduced or omitted, lower discarded quantities could be related to both increased food consumption and/or reduction in the amount of food prepared. Conclusions: High platform uptake supports the operational feasibility of implementing this combined programme in the participating schools. The observed differences are exploratory: the uncontrolled design, brief waste-measurement periods, and questionnaire attrition preclude causal attribution. Controlled studies with repeated measurements and longer follow-up are needed to evaluate effectiveness and transferability.

## 1. Introduction

The international community has recognized climate change as a real and major threat to human development [1]. Indeed, there is increasing apprehension surrounding the impact of climate change, especially regarding food security for the upcoming generations. Consequently, reducing food waste is considered one of the most prominent ways to improve food security [2]. Over the past few years, the issue of food waste has attracted the attention of various stakeholders such as policymakers, associations, industries, and academics [3]. This is because the Food and Agriculture Organization (FAO) estimates that 14% of globally produced food, valued at around $400 billion, is lost between harvest and retail sales [4], and an additional 17% is wasted at the consumer and retail levels [5]. The UNEP Food Waste Index Report for 2024 reveals that, in 2022, 60% of food waste was generated from households, 28% from food service, and 12% from retail [6]. Similarly, within the European Union (EU), households are responsible for over half of total food waste (54%), with 70% of this waste occurring at the household, food service, and retail levels [7]. In advanced economies, most of the food waste occurs at the later stages of the food chain, and it is mainly due to behavioral issues [8,9].

The school catering service is one of the areas where food waste is generated. Thus, understanding the potential determinants of food waste in school canteens is important for multiple reasons: these settings confer an ideal scenario to study food systems at the consumer level, and school canteens represent highly controlled environments where educational initiatives can be effectively implemented [10]. Accordingly, school catering can contribute to environmental protection by promoting sustainable food production practices. In Europe, policies such as Green Public Procurement [11,12] encourage reducing the environmental impact of public supply chains and advocate for enhancing public service quality by achieving economic, environmental, and social benefits. Nonetheless, studies conducted in Italian school settings have reported substantial levels of plate waste, particularly for side dishes [3,13]. Indeed, nutritional losses due to waste have been shown to affect key nutrients such as fiber, folate, and calcium, with energy losses ranging from 26% to 36% of total meal content [14]. Moreover, plate waste has been estimated to contribute significantly to both the environmental impact and economic cost of school meals, accounting for up to 28% of the carbon footprint and up to 10% of the total meal price [15].

School food procurement plays a crucial role in providing students with meals that are both energetically and nutritionally appropriate, supporting their growth, academic performance, and the development of healthy eating habits into adulthood [16]. The brain develops rapidly during early childhood, making it particularly vulnerable to nutritional deficiencies [17]. As such, a healthy diet during preschool and school years is essential for supporting cognitive and physical development [18], while also fostering lasting dietary habits and attitudes that benefit long-term health [19,20,21,22]. Food choices are shaped by both intrinsic factors (e.g., taste, smell, and appearance) and extrinsic factors (e.g., socioeconomic status, health awareness, and family routines) [23], alongside cognitive-behavioral elements such as explicit and implicit attitudes. These attitudes, whether informed by nutritional knowledge or emotional responses to food, significantly influence eating behavior [24,25,26]. Environmental exposure, through education and passive learning, may shape these attitudes and behaviors [27], though limited research has examined how such factors affect food preferences, waste, and family dynamics [26]. While evidence links poor diet to lower socioeconomic status [28] and extensive literature exists on children’s eating behavior [26,29,30], greater focus is needed on the broader influences that shape these behaviors. Overall, diet is a critical protective factor for healthy development and disease prevention. Promoting a balanced and sustainable food environment requires the coordinated efforts of individuals, families, and institutions [31].

In this context, the present report aims to provide a descriptive account of a pilot school-based intervention that introduced a digital tool for planning lunch menus and portion sizes within a primary and preschool setting. The implementation objectives were to integrate the software into routine school meal-management procedures, deliver the educational workshops and collect food-waste measurements and questionnaires before and after implementation. Accordingly, the report seeks to (i) document the implementation process and the feasibility of integrating new software, and describe platform uptake and use, (ii) present preliminary observations on food-waste levels before and after the intervention period, and (iii) explore children’s and parents’ dietary habits, food evaluations, food-waste attitudes and perceptions of inclusivity and involvement.

## 2. Materials and Methods

### 2.1. Study Design

**Figure 1:**
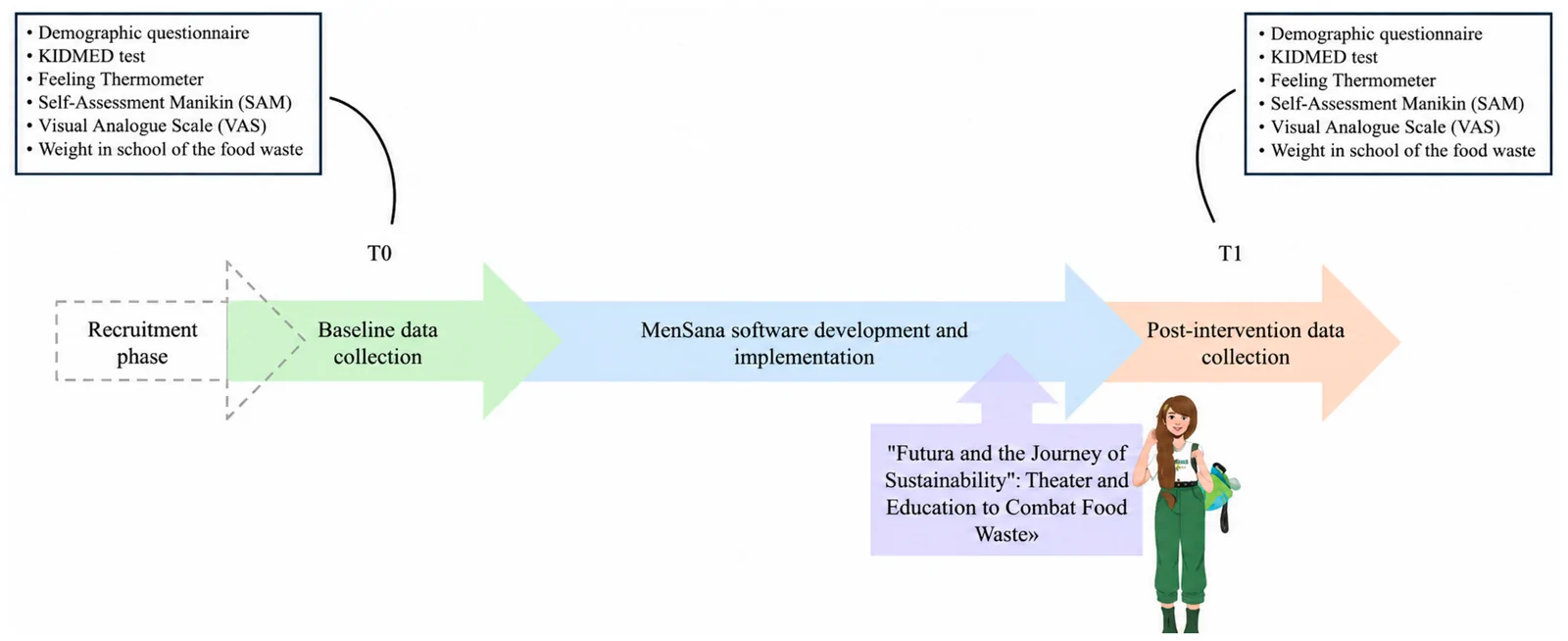
Project Timeline.

This study adopted an uncontrolled pre-post pilot study design comprising the following stages (see Figure 1): (a) recruiting preschool and primary schools in a local municipality (Borgarello, Pavia) and obtaining consent for participation in the study; (b) collecting baseline data (T0) by means of specific questionnaires for parents and children and collecting measurements on the weight of the food waste in the school canteens by a calibrated scale [32]; (c) developing and implementing the use of a new software “MenSana” (Version 1.0) for optimising meal preparation; (d) performing the “Futura” educational initiative which included a workshop for children and the realisation of audiovisual material; (e) re-administering questionnaires to parents and children and re-weighing the food waste at school (T1).

### 2.2. Study Population

**Figure 2:**
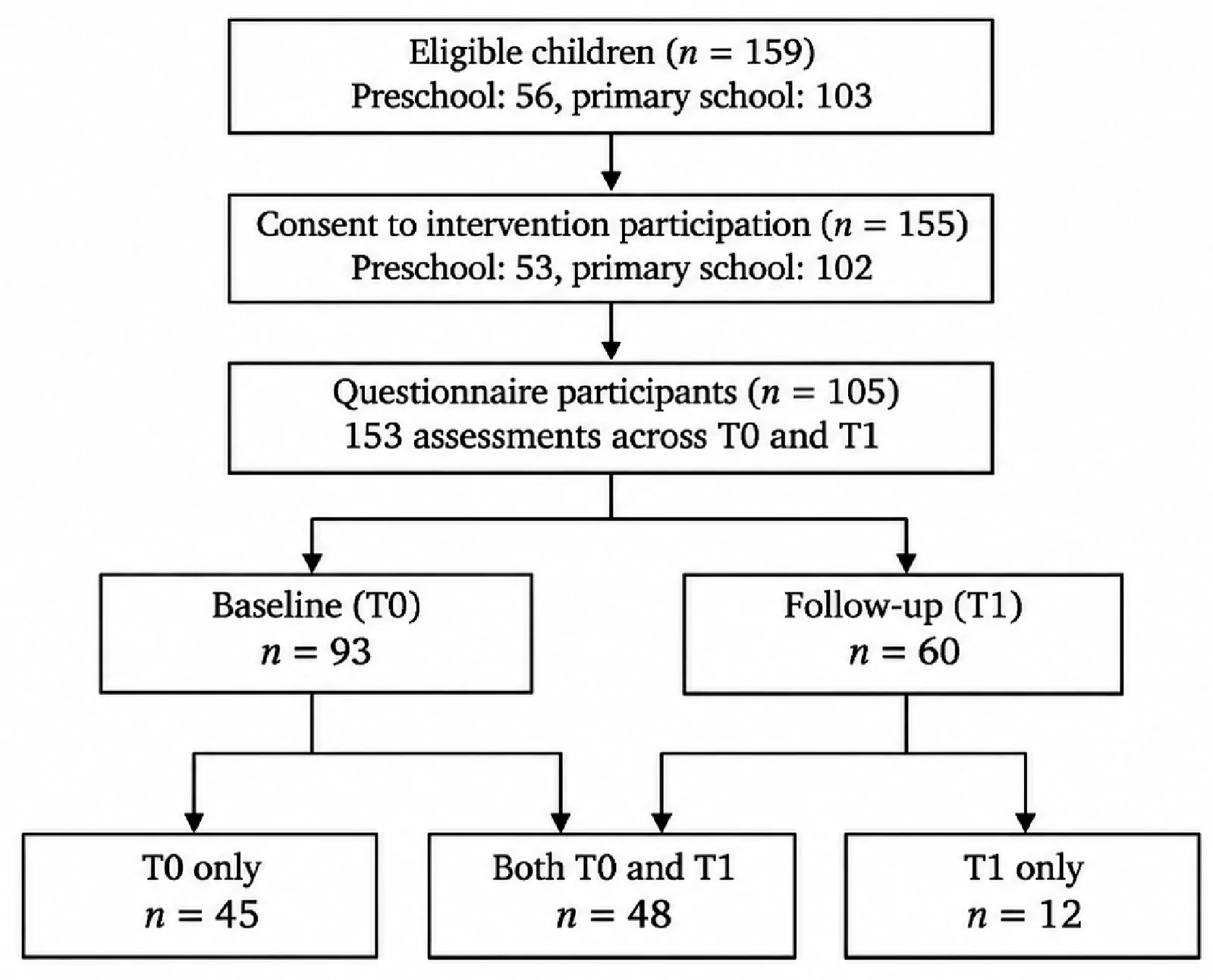
Participant flow through recruitment and questionnaire assessment. T0: baseline; T1: follow-up. Overall, 105 children completed 153 questionnaire assessments, including 48 children assessed at both time points.

All children enrolled in the participating schools were eligible to take part in the school-based activities. The school population included 159 children aged 3–11 years (103 primary school children and 56 preschool children). Questionnaire data were available for 105 children, comprising 153 parent–child questionnaire assessments: 93 at baseline (T0) and 60 at follow-up (T1). Of these, 48 contributed assessments at both time points, 45 at baseline only, and 12 at follow-up only. Thus, 48 of the 93 baseline participants (51.6%) contributed follow-up data. Families received repeated invitations to complete the follow-up questionnaire; however, reasons for non-participation were not recorded. Participant flow is presented in Figure 2. As for the intervention, the total number of participants who consented to participate was 155 (102 primary students; 53 preschool students). The protocol was reviewed and approved by the Ethical Committee of the Psychology Section, Department of Brain and Behavioral Sciences of the University of Pavia. Informed consent for children’s participation was obtained from their parents or legal guardians before data collection.

### 2.3. Interventions

Before the start of the intervention, the local Municipality, teachers, and parents were engaged through project presentation meetings, during which the research team illustrated the scientific rationale underlying the study and the planned procedures. All questions were addressed to boost informed participation and enhance family engagement. The interventions were conducted after the baseline data collection and consisted of the development and introduction of a new software, “MenSana”, and educational hands-on workshops for the students, “Futura lab”.

#### 2.3.1. MenSana Software

The software was developed to enable families to plan and customize children’s meals at school while also accounting for portion sizes. Through communication from the school, parents had the choice to switch from traditional meal service to the use of the software in app or desktop form. Meals were prepared by the same company, “Rosa dei Venti” (Pavia, Italy), which was included as a project partner. Alternative dishes were defined by nutritionists in agreement with local health institutions, in order to maintain the same balance of ingredients and nutrients as the original dishes. Families could log into the app to select lunch menus in advance, choosing between alternative dishes and different portion options: standard, half portion, tasting portion, or no portion. The system recorded allergens and dietary restrictions to support meal planning. It was encouraged that when planning the week’s meals, parents discuss and involve the child in the decision-making process. Developers also accounted for changes in the menu choice, allowing parents and children to change their choice 24 h before meal consumption. App usage data were automatically recorded, including the number of planned meals, portion choices, and frequency of use. The software was developed by “PlanEat”, a partner of the project—an online grocery and meal planning service focused on sustainability and reducing food waste. The intervention was based on the premise that freedom to choose portion sizes may raise awareness of food choices and their impact in terms of waste, inspire willingness to try new foods, and ultimately decrease food waste [33,34]. Additionally, a further objective of the platform was to align meal preparation with anticipated demand through advance menu and portion selection, thereby limiting the preparation of food that might otherwise remain unconsumed.

#### 2.3.2. “Futura” Lab

The educational initiative, ‘Futura—A Journey into Sustainability through Art’, was designed to complement the digital meal-planning platform by engaging children with activities related to food sustainability and food-waste prevention. The programme comprised two 50-min sessions per class, structured around an introductory story featuring the character Futura, artistic activities, and a closing discussion. Activities included dramatization, movement, songs and rhymes, drawing, and collage, addressing the value and origins of food, waste prevention, sustainable eating practices, and cultural diversity in food traditions. Content was adapted to school level: preschool activities emphasized play, songs and simple drawings, whereas primary-school activities involved more structured creative tasks and guided reflection. Sessions were led by a facilitator trained in acting, music and arts therapies, with teachers present. These activities were intended to encourage reflection and engagement with food sustainability; their specific contribution to the observed outcomes was not evaluated separately.

### 2.4. Data Collection and Measures

Online surveys were administered using LimeSurvey 3.28.7 [35] between December 2024 and February 2025 for the baseline data collection, and between June and July 2025 for the follow-up assessment. Plate-waste measurements followed a separate schedule, with assessment weeks in March (pre-intervention) and May 2025 (post-intervention), as detailed in Section 2.4.1. The survey was divided into two parts: one completed by the parent, composed of demographic questions (i.e., age, gender, education level, work hours), KIDMED questionnaire, and Visual Analogue Scale. The second part was completed by the child, following instructions suggesting that the adult should help the child to complete it if necessary; it was composed of a Self-Assessment Manikin Scale, Feeling Thermometer, and Kiddy-KINDL/Kid-KINDL questionnaire. Each section was tailored to the respondent’s role, and all questionnaires were either validated or custom-developed measures.

#### 2.4.1. Food Waste Measurement

Plate waste was measured during two five-day periods, 24–28 March 2025 (T0) and 19–23 May 2025 (T1). The scheduled menus were the same across the two periods, with alternative dishes available through the platform at T1 (Supplementary Table S1). Food waste was quantified after lunch, which was organized in specific time slots to allow accurate monitoring of waste and to correctly associate each child with their served meal. During the meal, each child received a tray containing the meal. To estimate food waste before and after the intervention, only leftovers on the plates were considered, defined as the amount of edible food served but not consumed [36], as these represent the main source of food waste in schools [37,38]. Waste per meal attendance was calculated by dividing total measured plate waste by cumulative daily attendance across the two schools. An exploratory estimate for platform users was obtained by subtracting the estimated contribution of non-users from total T1 waste, assuming that non-users generated the corresponding school- and day-specific baseline waste per attendance. Estimated waste attributable to platform users was divided by the corresponding number of platform-user meal attendances. Daily measurements, attendance counts and calculation details are provided in Supplementary Table S2. The protocol for measuring plate waste was defined by adapting methods described in previous studies [39,40,41,42]. Leftovers on each tray were separated by two trained cafeteria staff members and classified into “first course”, “second course”, and “side dishes” categories, before being weighed using a digital scale. Beverages were not included in the measurements, as only water was served. Menus were not differentiated for special diets, given the limited number of specific dietary requirements.

#### 2.4.2. KIDMED

The KIDMED (Mediterranean Diet Quality Index for children and adolescents) questionnaire was selected as a validated measure to assess dietary quality and adherence to the Mediterranean diet [43]. The KIDMED index is based on a 16-item scale covering dietary habits consistent or inconsistent with this dietary pattern. The questionnaire was administered in its Italian version [44] and completed by parents to ensure accurate reporting of their child’s dietary intake. Given its psychometric properties, relevance to Mediterranean cultural dietary norms, and widespread use in school-based nutritional studies, the KIDMED was deemed suitable for capturing the nutritional component of the present pilot study [45,46,47].

#### 2.4.3. Visual Analogue Scale

To assess parents’ emotional responses to food waste, a Visual Analogue Scale (VAS) was employed [48,49]. Parents were presented with three series of 5 images depicting examples of food waste and were asked to rate their reactions across three affective dimensions: (i) relevance, the perceived importance of the image; (ii) arousal, the intensity of emotional activation elicited; (iii) dominance, the extent to which the individual felt in control over the represented situation of waste.

Each dimension was rated on a continuous slider, ranging from 0 (e.g., not important/not arousing/not in control) to 10 (extremely important/highly arousing/completely in control). In addition, using the same VAS format, parents were asked to rate (1) the extent to which school food choices were perceived as respectful and inclusive toward them and their child (VAS Inclusivity)—“How respectful and inclusive do you think the school’s food choices are towards you and your child?”, and (2) the extent to which they and their child felt involved in school food-related decisions (VAS Involvement)—“How involved do you think you and your child have been in the school’s food choices?”. Responses were recorded on a continuous 0–10 slider, with higher scores indicating greater perceived inclusivity and involvement.

#### 2.4.4. Self-Assessment Manikin

To assess children’s emotional responses to food waste, the Self-Assessment Manikin (SAM) method was utilized [50,51,52]. This tool captures affective states along three key dimensions: valence (sad—happy), arousal (agitated—calm), and dominance (sense of control). Scores ranged from 1 to 9, with higher scores indicating happier emotional responses for valence, greater agitation for arousal, and a greater sense of control for dominance. Unlike adults, children did not use a visual analogue scale; instead, responses were recorded using a nine-category pictorial scale for each dimension. These pictorial representations were designed to be age-appropriate and intuitive, allowing children to easily associate their internal states with corresponding emotional expressions. Each child was presented with 5 images depicting food waste (different from the parents’ questionnaire) and asked to select the figure that best represented how they felt for each dimension. These ratings were used as exploratory measures of image-elicited emotional responses to food waste.

#### 2.4.5. Feeling Thermometer

**Figure 3:**
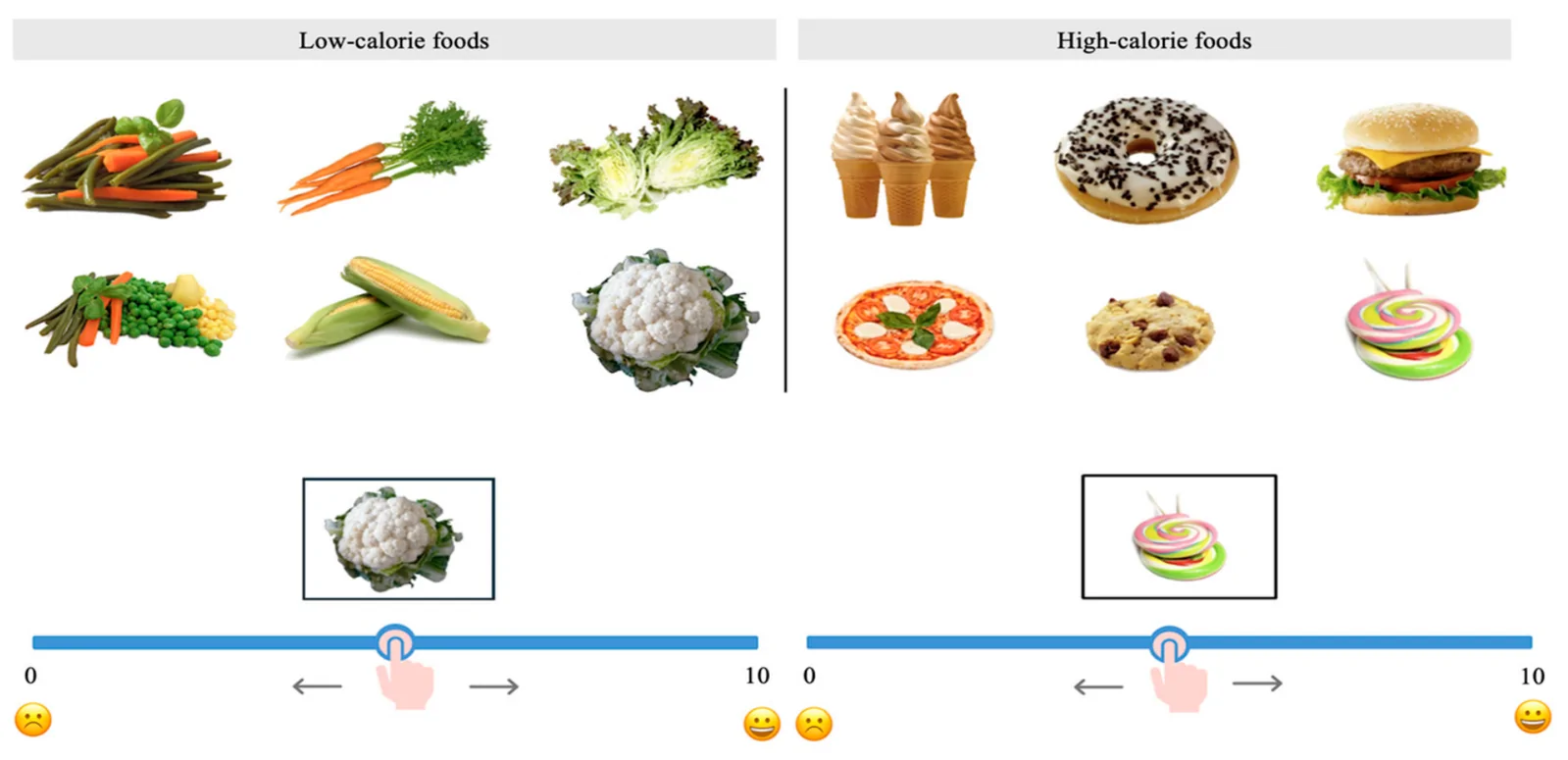
Food stimuli presented to the sample of children (**top** of panels) and examples of trials of the Feeling Thermometer (**bottom** of panels). The arrows indicate the direction in which the cursor could be moved by children.

Children’s emotional evaluations of different foods were assessed using the “Feeling Thermometer” [53,54,55]. They were shown a series of 12 images of low-calorie foods (e.g., carrots, peas) or high-calorie foods (e.g., donuts, hamburger), and for each item, they were asked to evaluate their emotional feeling using a visual thermometer with a slider, where 0 represented sad emotions, i.e., unpleasant emotional response, and 10 represented happiness, i.e., pleasant emotional response (see Figure 3). Stimuli were selected from the FoodCast research image database (FRIDa) [56] and FoodPics databases [57], and their selection was also informed by previous studies [53]. To aid comprehension and engagement, the scale was accompanied by illustrative sad and smiley faces at each end, serving as visual anchors.

#### 2.4.6. KINDL Health-Related Quality of Life Questionnaires

The KINDL is a validated psychological self-report instrument designed to assess health-related quality of life (HRQoL) in children and adolescents. The Kiddy-KINDL was administered to preschool children and Kid-KINDL to primary-school children. The preschool sample included children aged three years, below the standard age range of the Kiddy-KINDL child self-report version. The inclusion of children aged three reflected the class-based recruitment approach and was supported by previous research demonstrating the use of the Kiddy-KINDL self-report in children aged 3 to 5 years [58]. The instrument evaluates six core domains: (i) physical well-being; (ii) emotional well-being; (iii) self-esteem; (iv) family relationships; (v) peer relationships; (vi) school-related well-being [59]. The Kid-KINDL and Kiddy-KINDL questionnaires have been widely used in various cultural and linguistic contexts, including the Italian adaptation employed here [60].

### 2.5. Statistical Analysis

Descriptive statistics were used to summarise participant characteristics, questionnaire outcomes and platform-use records. Categorical variables were reported as frequencies and percentages, and continuous variables as means and standard deviations. The number of unique participants was distinguished from the number of collected questionnaires. Demographic characteristics were summarised once per participant, while baseline questionnaire summaries were restricted to T0 observations. Sample sizes were reported for each measure.

Exploratory baseline associations were examined using two-sided Pearson correlations between KIDMED scores and emotional evaluations of the high- and low-calorie food-image categories, perceived inclusivity, perceived involvement and KINDL scores. Each correlation used participants with available data for both variables.

To explore potential attrition bias, baseline characteristics were compared between participants who contributed follow-up questionnaire data and those who participated at baseline only. Participants with follow-up data only were excluded from these comparisons. Continuous variables were compared using Welch’s independent-samples *t*-tests, and categorical variables using Pearson’s chi-square tests. Mean differences, calculated as follow-up respondents minus non-respondents, were reported with unadjusted 95% confidence intervals. Detailed attrition-comparison results are provided in Supplementary Table S3.

Changes in responses to food-waste and food images between T0 and T1 were analysed in R software (version 4.5.2), with linear mixed-effects models (lme4 and lmerTest packages; REML estimation, Satterthwaite approximation for degrees of freedom), because each participant rated several images at both time points. Random intercepts for participants and for stimuli were therefore included to account for repeated ratings by the same participant and for variability across images; for the two parents’ items on perceived inclusivity and involvement, which were rated once per time point, only the random intercept for participants was included. Parents’ VAS and children’s SAM and Feeling Thermometer ratings were modelled separately. The models included 765 observations from 105 parents and 5 stimuli for each of the VAS arousal, relevance and dominance ratings; 153 observations from 105 parents for each of the inclusivity and involvement ratings; 650 observations from 96 children and 5 stimuli for each SAM dimension; and 1548 observations from 96 children and 12 stimuli for the Feeling Thermometer. To take into account differences between preschool and primary school, children’s models also included School Grade and Age as covariates. Fixed effects were tested with Type II Wald chi-square tests (car package), and estimated marginal means were obtained with the emmeans package. Post-hoc comparisons were performed with the phia package (testInteractions), applying Bonferroni correction to control for multiple comparisons. Effect estimates are reported as mean differences with 95% confidence intervals (CI); confidence intervals for post-hoc comparisons are Bonferroni-adjusted.

Because SAM, VAS and Feeling Thermometer ratings are discrete and bounded, the suitability of the linear models was evaluated by inspecting residual diagnostics (Q-Q plots, residuals versus fitted values, skewness and kurtosis; Supplementary Materials). As a sensitivity analysis, all models were also fitted as cumulative-link mixed models (ordinal package; logit link, Laplace approximation) with the same fixed and random effects and interactions, tested with likelihood-ratio tests. The main results reported are those of the linear mixed-effects models; the cumulative-link models are reported as sensitivity analyses (Section 3 and Supplementary Materials).

## 3. Results

### 3.1. “MenSana” Data and Food Waste

**Figure 4:**
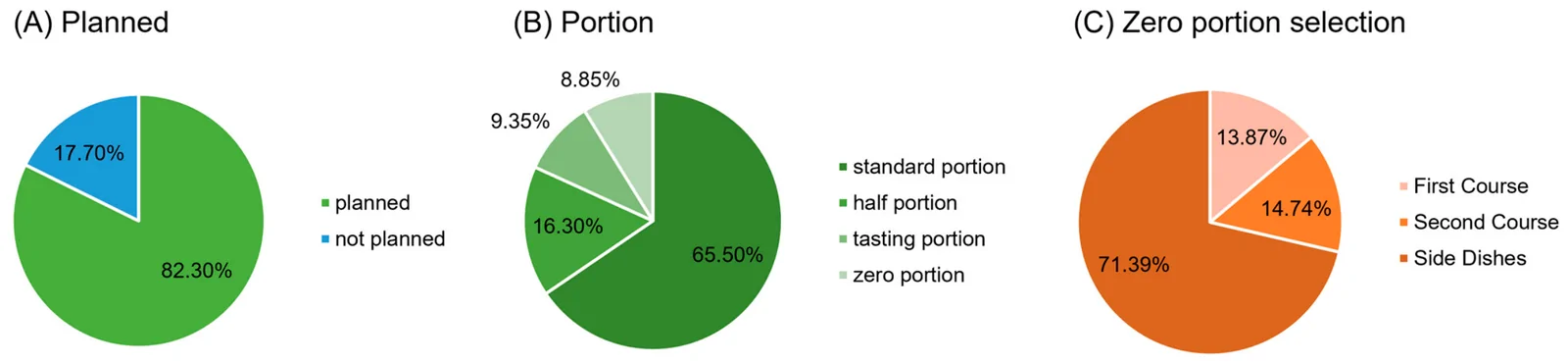
(**A**) Percentage distribution of planned meals. (**B**) Percentage of portion selection types for planned meals. (**C**) Percentage distribution of “zero portion” selections by meal course.

Of the 159 pupils enrolled in the schools (56 preschool, 103 primary), 155 registered on the MenSana platform, and 152 (95%) actively used it at least once. Engagement was high, with 90% of users customizing portion sizes during the intervention period. Overall, 82.3% of the meals served were pre-planned through the app during the period of use (Figure 4A). Among planned meals, 65.5% were selected in standard portions, 16.3% in half portions, 9.35% in tasting portions, and 8.85% in “zero portions”, where families chose not to receive the dish (Figure 4B). The customization option was frequently used for vegetables. For instance, green salad, steamed greens, and steamed carrots were among the most frequently selected items in tasting portions. Plate waste was measured during one week in March (T0) and one week in May (T1), using matched menus. Across all canteen attendees, waste decreased from 0.066 to 0.038 kg per meal attendance, an observed reduction of 42.1%. An additional calculation specific to platform users estimated waste at 0.032 kg per attendance at T1, corresponding to a 51.3% reduction relative to the overall baseline value. Daily measurements and details of the estimation procedure are provided in Supplementary Table S2. The side dishes, mainly vegetables, were the meals that were most often planned using the ‘zero portion’ option, accounting for 71.39% of all zero-portion selections (Figure 4C), and are typically the most wasted category in school cafeterias [13]. Over the eight-week monitoring period, the numbers of portions prepared were compared with the estimated numbers that would have been prepared without platform-based planning. The estimated differences were around 13% for first courses, around 13% for second courses and around 35% for side dishes (Supplementary Tables S4 and S5).

### 3.2. Descriptive Statistics

A total of 105 unique participants were included. Overall, 153 questionnaire assessments were completed: 93 at baseline (T0) and 60 at follow-up (T1). Of the total sample, 48 participants completed both time points (see Figure 2). Demographic characteristics were summarised once per participant across the unique participant sample (*N* = 105; Table 1). Baseline descriptives were computed to characterize the sample prior to the intervention. At T0, the mean KIDMED score was 5.35 (SD = 2.31), indicating overall a moderate level of adherence to the Mediterranean diet. Considering the cut-off scores proposed by the KIDMED index [61], 20 (21.5%) families reported poor adherence to the Mediterranean diet, 58 (62.4%) families had medium adherence and 15 (16.1%) families had high adherence. Among parents, mean VAS ratings of food-waste images on relevance, arousal and dominance were 8.67 (SD = 2.44), 7.77 (SD = 2.71), and 6.77 (SD = 2.86), respectively. Instead, mean SAM scores on valence, arousal and dominance for children’s emotional responses to food-waste images were 2.24 (SD = 1.30), 5.23 (SD = 2.28), and 5.20 (SD = 2.44), respectively. The mean KINDL score was 77 (SD = 12), ranging from 29.2 to 100, indicating generally moderate-to-high levels of well-being. The mean Feeling Thermometer score was 6.45 (SD = 1.53). Children gave higher Feeling Thermometer ratings to the presented high-calorie food images (M = 7.68, SD = 1.72) than to the presented low-calorie food images (M = 5.21, SD = 2.58).

### 3.3. Baseline Analyses

Baseline characteristics were compared between participants with follow-up data (*n* = 48) and those with baseline data only (*n* = 45; Supplementary Table S3). Comparisons indicated lower parental VAS dominance and perceived involvement among subsequent follow-up respondents. No other comparisons had *p*-values below 0.05. Correlation analyses were conducted using R [62] to examine associations between baseline (T0) measures of dietary adherence, food emotional evaluation, inclusivity perceptions, and child well-being. KIDMED scores were positively associated with emotional evaluation for low-calorie foods (r = 0.377, *p* < 0.001), whereas no significant association was observed with emotional evaluation for high-calorie foods (r = −0.067, *p* = 0.678). Additionally, KIDMED scores were positively correlated with VAS perceived inclusivity scores of school food choices (r = 0.343, *p* < 0.001), while the association with VAS perceived involvement scores in food-related decisions was not statistically significant (r = 0.149, *p* = 0.153). A significant positive association was observed between KIDMED scores and child well-being (KINDL scores) (r = 0.240, *p* = 0.033).

### 3.4. Exploratory Changes in Responses to Food-Waste Images

**Figure 5:**
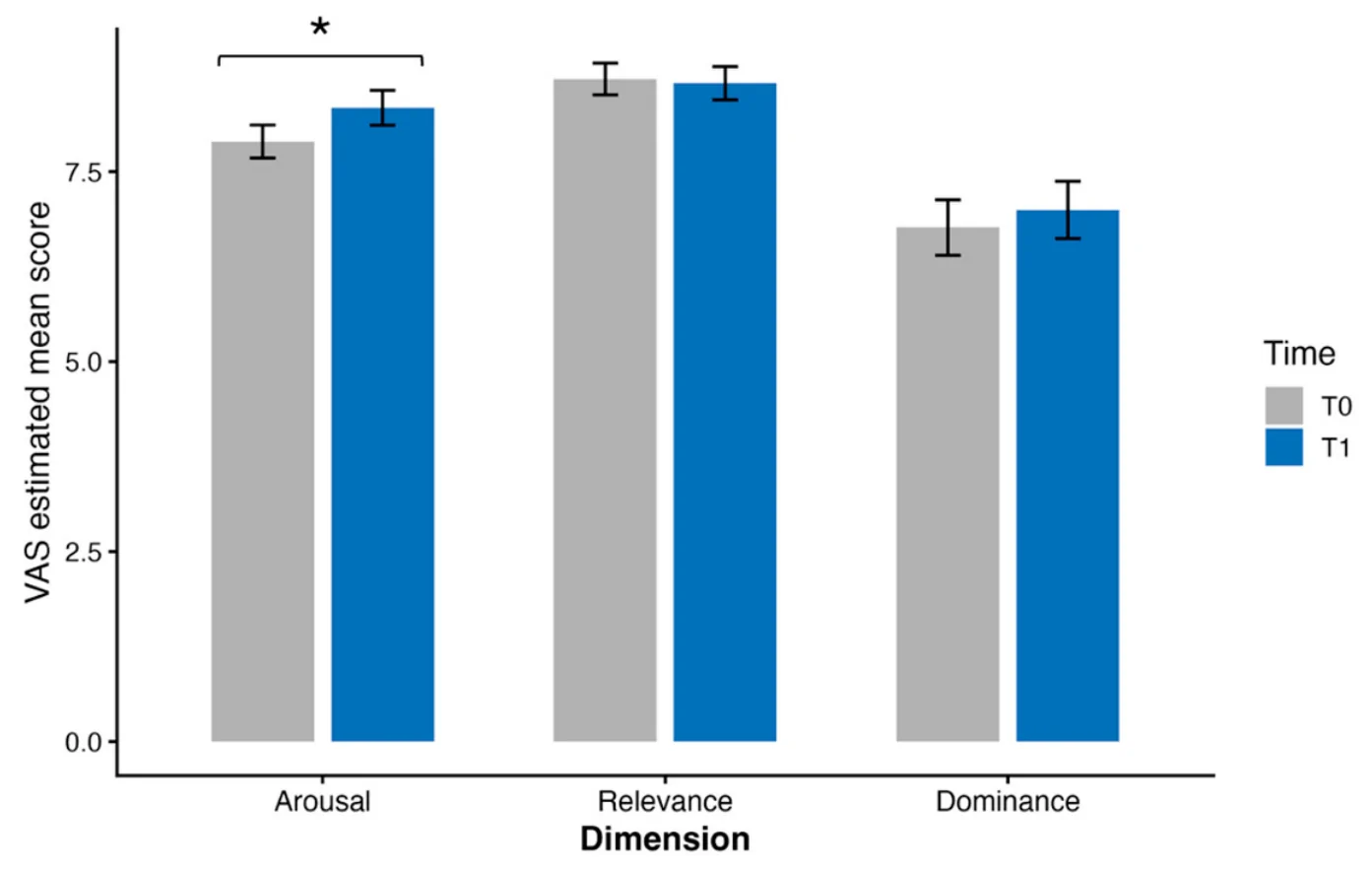
Estimated marginal means of VAS scores for each dimension at T0 (grey) and T1 (blue), derived from the linear mixed-effects models. Bars represent estimated marginal means and error bars indicate ± 1 standard error (SE). Brackets with an asterisk indicate a significant Time effect.

Ratings of the food-waste images given by parents (VAS) and children (SAM) were compared between T0 and T1. Results showed a significant effect of Time on parents’ VAS arousal ratings (χ^2^(1) = 10.31, *p* = 0.001), with higher scores at T1 (M = 8.34, SE = 0.23, 95% CI [7.88, 8.80]) compared to T0 (M = 7.90, SE = 0.21, 95% CI [7.47, 8.33]; mean difference T1 − T0 = 0.44, 95% CI [0.17, 0.72]), suggesting an increased level of activation in response to food waste stimuli. For VAS relevance, there was no significant effect of Time (χ^2^(1) = 0.23, *p* = 0.634; mean difference T1 − T0 = −0.06, 95% CI [−0.28, 0.17]; see also the sensitivity analysis in Supplementary Materials). Similarly, for VAS dominance, there was no significant effect of Time (χ^2^(1) = 1.90, *p* = 0.168; mean difference T1 − T0 = 0.23, 95% CI [−0.10, 0.56]) (Figure 5).

**Figure 6:**
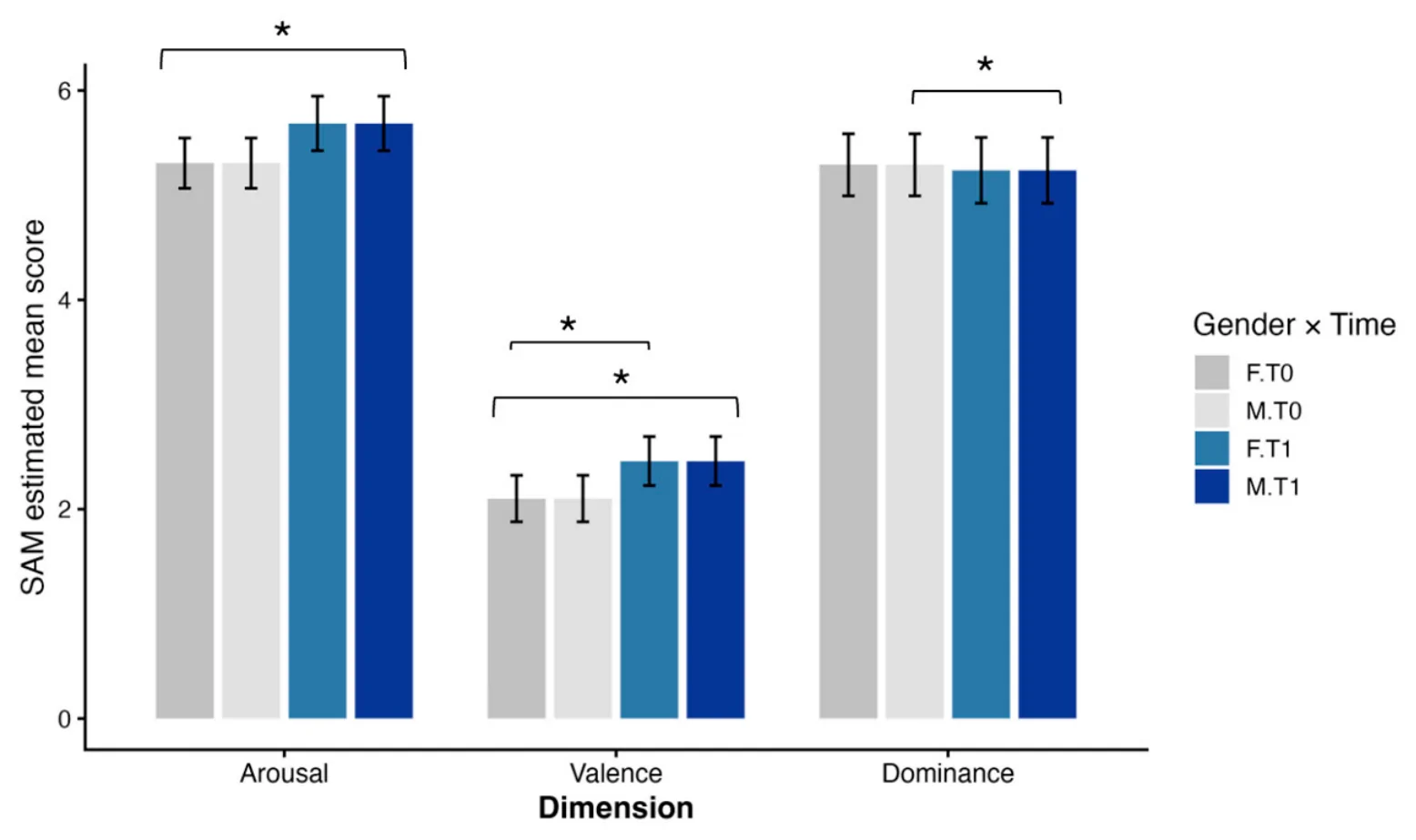
Estimated marginal means of children’s SAM (Self-Assessment Manikin) scores for each dimension, by gender and time point, derived from the linear mixed-effects models. Bars represent estimated marginal means for girls (F) and boys (M) at T0 (grey) and T1 (blue). Error bars indicate ± 1 standard error (SE). Long brackets with asterisks indicate a significant effect of Time in the models on Arousal and Valence; short brackets with asterisks indicate a significant effect of the time x gender interaction, highlighting significant post-hoc comparisons between T0 and T1 in females for Valence score and between T0 and T1 in males for Dominance score.

Regarding children’s SAM arousal ratings, results showed a significant effect of Time (χ^2^(1) = 6.62, *p* = 0.010), with higher scores at T1 (M = 5.21, SE = 0.31, 95% CI [4.59, 5.83]) compared to T0 (M = 4.75, SE = 0.29, 95% CI [4.16, 5.33]), corresponding to a mean difference of 0.46 (95% CI [0.11, 0.82]). There was no significant effect of Gender (χ^2^(1) = 3.79, *p* = 0.052) and no significant Time × Gender interaction (χ^2^(1) = 0.004, *p* = 0.949). The effect of School Grade was significant (χ^2^(1) = 6.58, *p* = 0.010), with higher arousal in primary school children than in preschoolers (b = 1.80, SE = 0.70, 95% CI [0.42, 3.17]), whereas there was no significant effect of Age (χ^2^(1) = 0.64, *p* = 0.423) (Figure 6).

For SAM valence, results showed a significant effect of Time (χ^2^(1) = 6.54, *p* = 0.011), with higher scores at T1 (M = 2.56, SE = 0.27, 95% CI [2.00, 3.12]) compared to T0 (M = 2.16, SE = 0.26, 95% CI [1.62, 2.70]). There was no significant effect of Gender (χ^2^(1) = 0.14, *p* = 0.713), School Grade (χ^2^(1) = 0.34, *p* = 0.561; b = −0.29, SE = 0.50, 95% CI [−1.26, 0.69]) or Age (χ^2^(1) = 0.02, *p* = 0.887). The Time × Gender interaction was significant (χ^2^(1) = 11.89, *p* < 0.001). Bonferroni-corrected post-hoc comparisons showed a significant increase between T0 and T1 among females (*p* < 0.001; Δ = 0.87, 95% CI [0.47, 1.27]; T0: M = 2.04, SE = 0.30, 95% CI [1.43, 2.65]; T1: M = 2.91, SE = 0.32, 95% CI [2.25, 3.56]), and no significant change among males (*p* = 1.000; Δ = −0.07, 95% CI [−0.43, 0.29]; T0: M = 2.28, SE = 0.28, 95% CI [1.69, 2.86]; T1: M = 2.20, SE = 0.30, 95% CI [1.59, 2.82]).

For SAM dominance, results showed no significant effect of Time (χ^2^(1) = 0.40, *p* = 0.526) or Gender (χ^2^(1) = 0.33, *p* = 0.563). The Time × Gender interaction was significant (χ^2^(1) = 10.03, *p* = 0.002). The effect of School Grade was significant (χ^2^(1) = 4.38, *p* = 0.036), with higher dominance in primary school children than in preschoolers (b = 1.65, SE = 0.79, 95% CI [0.11, 3.19]), whereas there was no significant effect of Age (χ^2^(1) = 1.07, *p* = 0.301). Bonferroni-corrected post-hoc comparisons showed a significant decrease between T0 and T1 among males (*p* = 0.020; Δ = −0.65, 95% CI [−1.14, −0.15]; T0: M = 5.21, SE = 0.41, 95% CI [4.38, 6.05]; T1: M = 4.57, SE = 0.43, 95% CI [3.70, 5.44]), and no significant change among females (*p* = 0.105; Δ = 0.54, 95% CI [−0.01, 1.10]; T0: M = 4.55, SE = 0.44, 95% CI [3.67, 5.43]; T1: M = 5.09, SE = 0.47, 95% CI [4.15, 6.03]). The post-hoc result in males was not confirmed by the ordinal model (see sensitivity analysis in Supplementary Materials) (Figure 6).

### 3.5. Changes in Perceived Inclusivity and Involvement

**Figure 7:**
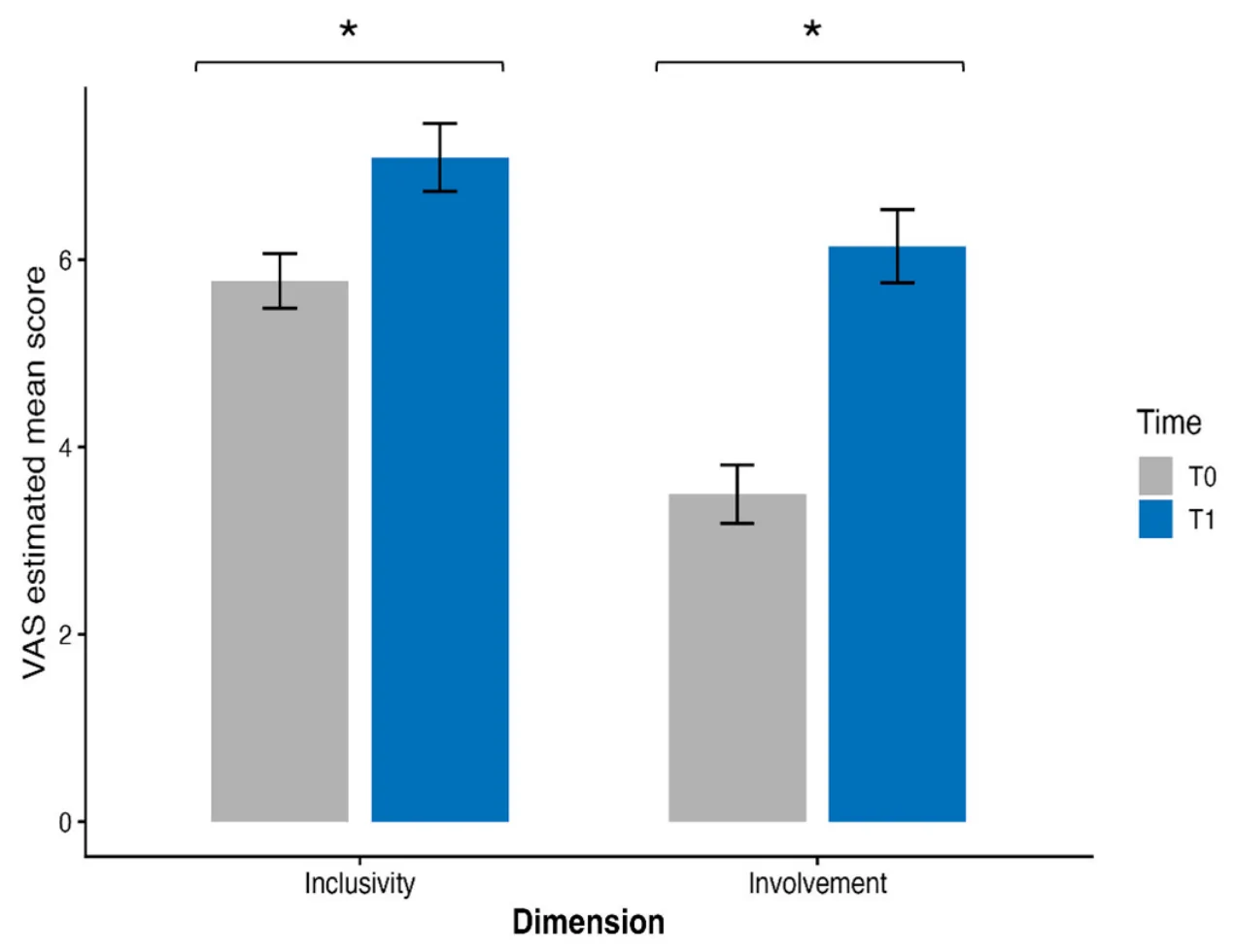
Estimated marginal mean scores for inclusivity and involvement at baseline (T0) and follow-up (T1), derived from linear mixed-effects models. Bars represent the model-estimated marginal mean scores for the two dimensions, inclusivity and involvement, measured using a Visual Analogue Scale (VAS) at baseline (T0, grey bars) and follow-up (T1, blue bars). Error bars indicate the standard errors (SE) of the estimated marginal means. Brackets and asterisks indicate a significant effect of time in both dimensions.

To examine changes in parents’ perceptions of inclusivity and involvement, VAS ratings were compared between T0 and T1. Results on perceived inclusivity showed a significant effect of Time (χ^2^(1) = 9.06, *p* = 0.003), with higher scores at T1 (M = 7.07, SE = 0.36, 95% CI [6.35, 7.78]) compared to T0 (M = 5.79, SE = 0.29, 95% CI [5.22, 6.37]; mean difference T1 − T0 = 1.27, 95% CI [0.43, 2.12]), suggesting an improvement in perceived inclusivity following the intervention. For perceived involvement, there was also a significant effect of Time (χ^2^(1) = 23.52, *p* < 0.001), with higher scores at T1 (M = 6.07, SE = 0.41, 95% CI [5.26, 6.87]) compared to T0 (M = 3.55, SE = 0.33, 95% CI [2.90, 4.19]; mean difference T1 − T0 = 2.52, 95% CI [1.48, 3.56]) (Figure 7).

### 3.6. Exploratory Food-Image Evaluations

To investigate children’s emotional evaluation of food, Feeling Thermometer scores were analysed as a function of Time, Food Category (high-calorie vs. low-calorie) and Gender. Results showed no significant effect of Time (χ^2^(1) = 1.84, *p* = 0.174), but a significant main effect of Food Category (χ^2^(1) = 21.35, *p* < 0.001) and Gender (χ^2^(1) = 4.79, *p* = 0.029). Furthermore, a significant Food Category × Gender interaction was observed (χ^2^(1) = 38.73, *p* < 0.001). No other interaction was significant (Time × Food Category: χ^2^(1) = 0.68, *p* = 0.410; Time × Gender: χ^2^(1) = 0.57, *p* = 0.449; Time × Food Category × Gender: χ^2^(1) = 2.15, *p* = 0.142). There was no significant effect of School Grade (χ^2^(1) = 2.85, *p* = 0.091) or Age (χ^2^(1) = 2.79, *p* = 0.095).

**Figure 8:**
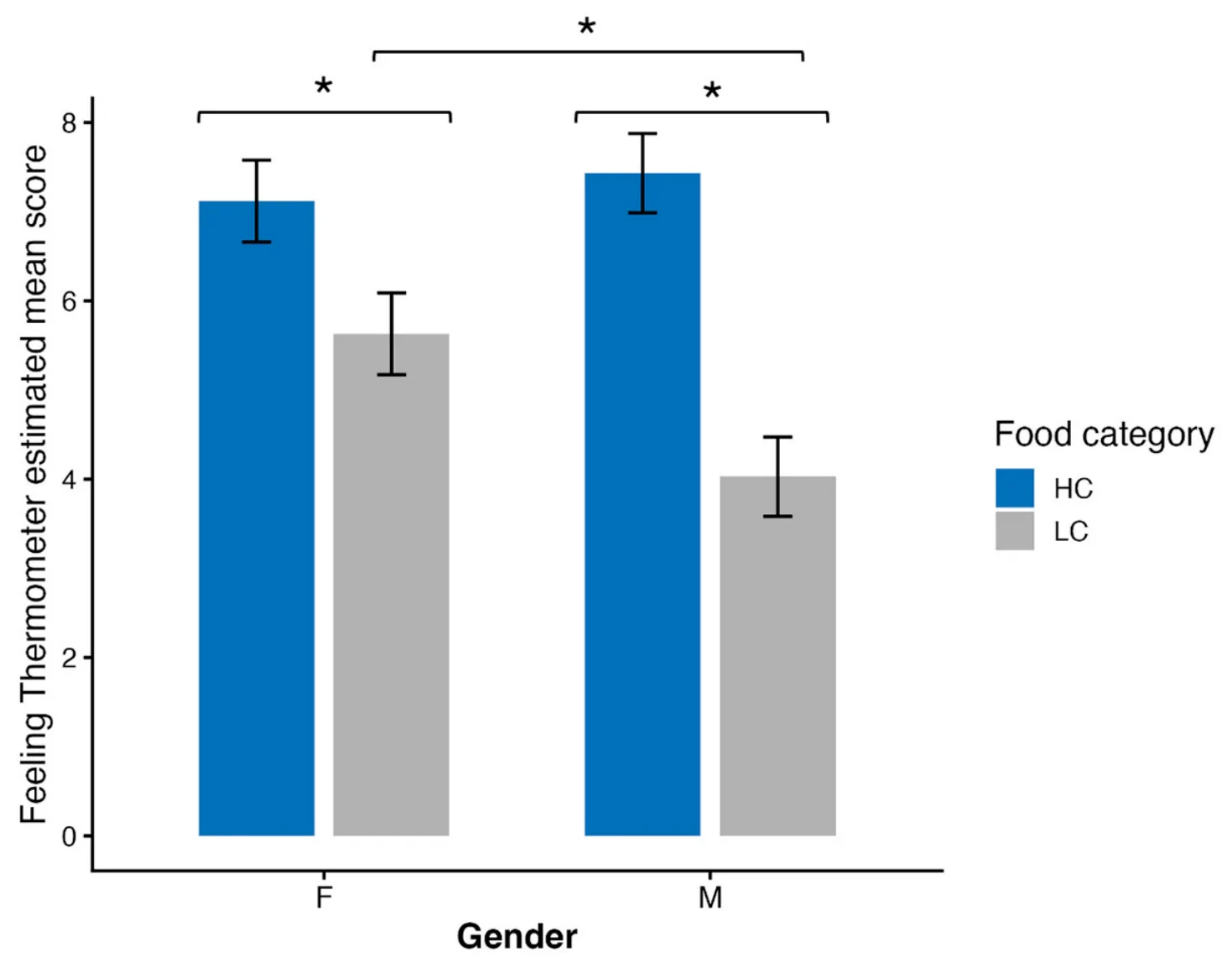
Estimated marginal means of scores for high-calorie (HC) and low-calorie (LC) foods by gender derived from linear mixed-effects models. Error bars indicate ± 1 standard error (SE). Brackets and asterisks indicate significant differences between HC and LC foods in both male and female samples, and significant differences in LC evaluation between females and males.

Post-hoc comparisons showed significant differences between HC and LC food in both females (χ^2^(1) = 6.94, *p* = 0.017; Δ = 1.49, 95% CI [0.26, 2.72]; HC: M = 7.12, SE = 0.46, 95% CI [6.16, 8.07]; LC: M = 5.63, SE = 0.46, 95% CI [4.67, 6.59]) and males (χ^2^(1) = 37.21, *p* < 0.001; Δ = 3.40, 95% CI [2.18, 4.63]; HC: M = 7.43, SE = 0.45, 95% CI [6.50, 8.37]; LC: M = 4.03, SE = 0.45, 95% CI [3.09, 4.96]), with higher scores for HC than LC food and a larger HC–LC difference in males. Moreover, for LC food, females showed higher scores than males (χ^2^(1) = 25.26, *p* < 0.001; Δ = 1.60, 95% CI [0.97, 2.23]), whereas no significant gender difference emerged for HC food (χ^2^(1) = 0.97, *p* = 0.650; Δ = −0.31, 95% CI [−0.94, 0.32]) (Figure 8).

### 3.7. Sensitivity Analyses with Cumulative-Link Mixed Models

Because outcomes were collected on different rating scales (VAS, SAM, Feeling Thermometer), we inspected the residuals of every linear mixed model (Q-Q plots and residuals-vs-fitted plots, Supplementary Figures S1–S9). Shapiro–Wilk tests were significant for all models (all *p* < 0.01). Visual inspection of the Q–Q plots revealed varying degrees of departure from normality across models, with some showing substantial deviations and others displaying approximately normal residual distributions. The parents’ VAS relevance clearly showed a non-normal distribution of residuals (skewness = −1.63, kurtosis = 13.79), reflecting a marked ceiling effect, and, to a lesser extent, children’s SAM valence (skewness = 1.15, kurtosis = 6.35). Given these findings, cumulative-link mixed models (CLMMs) were additionally fitted as a sensitivity analysis to assess the robustness of the results. The main findings were confirmed: the increases in perceived inclusivity and involvement; the increase in children’s SAM arousal and the effect of school grade on arousal; the Time × Gender interaction on SAM valence (increase in females, no change in males); and the Food Category × Gender interaction on the Feeling Thermometer. Results were only partly confirmed for parents’ VAS arousal (same direction, but marginal; *p* = 0.055) and for children’s SAM dominance, where the Time × Gender interaction was significant, but the post-hoc changes were not robust (decrease in males no longer significant; increase in females borderline after Bonferroni correction) and the school grade effect was reduced to a trend. For parents’ VAS relevance, the two approaches diverged: the linear model detected no change in mean ratings, whereas the cumulative-link model indicated a shift of the distribution towards lower ratings at T1, so this result should be interpreted with caution.

## 4. Discussion

Previous studies indicate that promoting food education and sustainability in schools can increase the knowledge and attitudes of children and families toward healthy eating, foster inclusivity, and reduce food waste [63,64,65]. These findings crucially highlight the relevance of improving school food policies, given that school cafeterias represent a significant source of nutritional, economic, and environmental loss [13].

This uncontrolled pre–post pilot study documented the implementation of a programme combining digital meal planning with educational workshops in a preschool and a primary school. It explored operational feasibility and preliminary changes in measured plate waste, parent-reported Mediterranean diet adherence, emotional evaluations of food and food-waste images, and perceived inclusivity and involvement. The findings provide information about implementation in this setting, while the absence of a concurrent comparison group limits interpretation of changes over time.

The study evaluated a multicomponent programme combining digital meal planning, parental involvement, portion customization and educational workshops. The contributions of these components could not be separated, and the uncontrolled design also prevents causal attribution of the observed changes to the programme as a whole.

Emotional responses to food-waste images were included to explore an affective aspect of attitudes towards food waste within the broader context of sustainability, drawing on previous research examining affective evaluations of food-wasting behaviour [51]. Regarding these attitudes, the results show that both parents and children exhibited increased emotional arousal in response to images of food waste between T0 and T1, indicating a difference in reported emotional activation between assessments. Specifically, children showed increased valence (i.e., sad—happy dimension) at T1 compared to T0, with the time of assessment interacting with gender, as the valence specifically increased in female students. The dominance dimensions revealed different patterns in males and females, but neither showed robust significant differences between T0 and T1. These exploratory findings suggest a change in children’s self-reported emotional responses to food-waste images following the intervention. Measured plate waste per meal attendance was approximately 42.1% lower during the follow-up assessment week than during the baseline assessment week. This represents a descriptive difference between two observation periods. Platform records also documented families’ menu and portion selections, including customization of vegetable side dishes. These observations suggest a possible role for aligning food provision with requested portions. Portion customization also raises questions about food exposure and nutritional intake. During project meetings, the tasting option was encouraged to provide an opportunity to try less familiar or less preferred foods, consistent with the rationale of repeated exposure discussed in previous research [66,67,68,69,70]. However, selection of a tasting portion does not establish that the child tasted or accepted the food. Reducing food provision through demand-informed preparation was itself an intended component of the intervention’s waste-prevention strategy. A reduction in discarded food arising from better alignment between preparation and demand would therefore be relevant to this objective, even without increased consumption. However, this potential operational benefit should be distinguished from improvements in dietary intake or nutritional adequacy. Without measurements of the quantities served and consumed, lower plate waste cannot establish increased consumption. The implications of reducing or omitting vegetable portions therefore require further investigation.

Platform-use records indicate substantial family engagement with digital meal planning in this setting. These findings provide descriptive support for the operational feasibility of implementing the platform within existing school catering arrangements. However, uptake and use alone do not establish usability, acceptability, or increased awareness of food choices.

Regarding perceived inclusivity, an improvement was observed from T0 to T1 in both overall inclusivity and perceived involvement, while these findings reflect respondents’ perceptions rather than objective changes in service equity or accessibility, they provide preliminary evidence of improvement in how the service was experienced. Given the uncontrolled design and differences in questionnaire participation across assessments, these findings should be interpreted cautiously and considered exploratory.

Interesting data emerged from children’s food evaluation. Consistently with previous evidence, children rated the presented high-calorie food images more positively than the low-calorie food images. Moreover, the difference in emotional ratings between the high- and low-calorie food images was larger in boys than in girls. Girls also gave higher ratings to the low-calorie food images than boys [71,72]. It is important to note that the food stimulus sets were not matched for other potentially relevant characteristics, including food type and degree of processing. In particular, the high-calorie set predominantly depicted processed or prepared foods, whereas the low-calorie set predominantly depicted vegetables. Consequently, differences in emotional ratings between the sets cannot be attributed specifically to calorie content and may also reflect responses to these other characteristics or differences in familiarity with the depicted foods. Future studies could use stimulus sets balanced across these characteristics to examine the contribution of calorie content more specifically.

Overall, children’s food-image ratings showed no clear change over time and remained predominantly oriented toward high-calorie foods. This pattern was also reflected in the frequent selection of the “zero portion” option for vegetables and side dishes, consistent with previous findings from Italian and international school settings [8,15]. However, the observed differences in ratings between the selected food-image categories should not be interpreted as evidence of corresponding differences in actual food selection or consumption.

At baseline, KIDMED scores indicated moderate adherence to the Mediterranean diet overall. Higher parent-reported adherence was associated with more positive emotional evaluations of the selected low-calorie food images and greater perceived inclusivity. These exploratory associations describe relationships between reported dietary habits, emotional food evaluations and perceptions of school catering.

Although these outcomes capture different aspects of eating behaviour and cannot be directly linked at the individual level, their combined pattern suggests that improvements in food waste may occur even in the absence of measurable changes in children’s emotional evaluations of the selected food images. Within the exploratory nature of this pilot study, these findings support the potential value of digital meal planning and portion personalization as strategies for reducing school food waste. The findings support further investigation of the combined programme in controlled studies.

## 5. Limitations and Future Research

Several limitations should be considered. This uncontrolled pre-post pilot involved a preschool and a primary school in one municipality, limiting generalisability. Changes between assessments cannot be separated from maturation, seasonality, developments over the school year, or other contextual influences. The multicomponent design also prevents the contributions of digital meal planning, parental involvement, portion customization and educational activities from being distinguished.

Plate waste was measured during only one week at each assessment. Although the menus were the same in the observed weeks, these short periods of data collection may not represent usual waste levels and may be affected by attendance and day-to-day variation. Moreover, the availability of smaller or omitted portions at follow-up may have reduced the amount of food served. Without corresponding served-mass and consumption measurements, the observed reduction in discarded food cannot establish increased consumption or improved nutritional intake.

Incomplete questionnaire follow-up introduces a risk of selection and attrition bias. Participants responding at follow-up may differ from those who did not respond, and changes in the composition of respondents may influence comparisons over time. Mixed-effects models can incorporate incomplete repeated observations under assumptions about missingness, but they do not eliminate this potential bias. The modest sample and multiple exploratory comparisons also warrant cautious interpretation.

The measures captured distinct constructs. KIDMED assessed parent-reported Mediterranean diet adherence, while the Feeling Thermometer and SAM assessed emotional responses to selected images. These ratings do not directly establish food choices, consumption or general sustainability attitudes. The food-image categories also differed in characteristics beyond calorie content, as discussed above. Children’s differing ages and developmental abilities may have influenced their understanding and use of the scales.

Moreover, another possible limitation of the present study concerns the assessment of children’s food preferences. Specifically, the Feeling Thermometer rating scale was used, in which children were asked how they felt when looking at a given food, using a response scale ranging from a sad face (0) to a happy face (10). Although this instrument allowed for an intuitive and age-appropriate assessment of children’s emotional reactions to foods, it may have partially overlapped emotional responses with actual food preferences. In other words, the measure may have conflated the constructs of “liking” and “wanting” with the more general emotional state elicited by food stimuli. Future studies could therefore benefit from the inclusion of an additional control measure specifically aimed at assessing food preference or liking/wanting, in order to more clearly disentangle emotional evaluation from hedonic preference. In this regard, the use of implicit measures such as the Implicit Association Test (IAT) for children could be particularly useful. Such approaches would help reduce potential biases related to awareness or social desirability and provide a more robust assessment of children’s underlying food preferences.

Future evaluations should document intervention delivery, participation, adaptations and contextual factors alongside outcomes. A controlled evaluation of the complete programme would assess its combined effect; distinguishing the contributions of individual components would require a design that specifically compares those components.

## 6. Conclusions

This pre–post pilot documented the implementation of a programme combining digital meal planning, family participation and sustainability workshops in a preschool and a primary school. Substantial platform uptake provided descriptive support for operational feasibility in this setting, and measured plate waste was lower during the follow-up observation week. Questionnaire outcomes were exploratory and concerned reported dietary adherence, emotional evaluations and perceived inclusivity and involvement.

The absence of a concurrent comparison group, limited waste-monitoring periods and incomplete questionnaire follow-up prevent conclusions about effectiveness. Lower plate waste may also reflect reduced food provision and does not establish improved consumption or dietary quality. These findings can inform further development and controlled evaluation of the programme across additional schools with longer follow-up, to foster research and interventions promoting health, sustainability and inclusivity.

## Figures and Tables

**Table 1:** Demographic characteristics were computed on the unique participant sample (*N* = 105).

	Value
Parent age (years)	M = 41.8, SD = 5.51 (Range 25–59)
Child age (years)	M = 7.46, SD = 2.38 (Range 3–11)
Child sex	Female 48 (45.7%); Male 53 (50.5%); Missing 4 (3.8%)
Diet type	Mediterranean diet: 100 (95.2%); Other/Vegetarian: 1 (1%); Other: 4 (3.8%)
Nationality	Italian: 99 (94.3%); European: 4 (3.8%); Extra-European: 2 (1.9%)

## Data Availability

The raw data supporting the conclusions of this article will be made available by the authors on request.

## Supplementary Materials

The following supporting information can be downloaded at: https://www.mdpi.com/article/10.3390/nu18213700/s1. Table S1: School menus and platform alternatives; Table S2: Daily plate-waste measurements, meal attendances and waste calculations; Table S3: Baseline characteristics according to follow-up questionnaire participation; Table S4: Sample-week food-preparation records; Table S5: Eight-week food-preparation records; Figure S1: Model diagnostic plots: QQ-plot of VAS arousal residuals to assess normality, and residuals versus fitted values plot to evaluate homoscedasticity and model adequacy; Figure S2: Model diagnostic plots: QQ-plot of VAS relevance residuals to assess normality, and residuals versus fitted values plot to evaluate homoscedasticity and model adequacy; Figure S3: Model diagnostic plots: QQ-plot of VAS dominance residuals to assess normality, and residuals versus fitted values plot to evaluate homoscedasticity and model adequacy; Figure S4: Model diagnostic plots: QQ-plot of SAM arousal residuals to assess normality, and residuals versus fitted values plot to evaluate homoscedasticity and model adequacy; Figure S5: Model diagnostic plots: QQ-plot of SAM valence residuals to assess normality, and residuals versus fitted values plot to evaluate homoscedasticity and model adequacy; Figure S6: Model diagnostic plots: QQ-plot of SAM dominance residuals to assess normality, and residuals versus fitted values plot to evaluate homoscedasticity and model adequacy; Figure S7: Model diagnostic plots: QQ-plot of VAS inclusivity residuals to assess normality, and residuals versus fitted values plot to evaluate homoscedasticity and model adequacy; Figure S8: Model diagnostic plots: QQ-plot of VAS involvement residuals to assess normality, and residuals versus fitted values plot to evaluate homoscedasticity and model adequacy; Figure S9: Model diagnostic plots: QQ-plot of Feeling Thermometer residuals to assess normality, and residuals versus fitted values plot to evaluate homoscedasticity and model adequacy; supplementary methods and results for the cumulative-link mixed-model sensitivity analyses.

## References

[1] Garnett T. (2011). Where Are the Best Opportunities for Reducing Greenhouse Gas Emissions in the Food System (Including the Food Chain)?. Food Policy.

[2] Kummu M., De Moel H., Porkka M., Siebert S., Varis O., Ward P.J. (2012). Lost Food, Wasted Resources: Global Food Supply Chain Losses and Their Impacts on Freshwater, Cropland, and Fertiliser Use. Sci. Total Environ..

[3] Lagorio A., Pinto R., Golini R. (2018). Food Waste Reduction in School Canteens: Evidence from an Italian Case. J. Clean. Prod..

[4] FAO (2019). The State of Food and Agriculture 2019.

[5] United Nations Environment Programme (2021). Food Waste Index Report 2021.

[6] United Nations Environment Programme (2024). Food Waste Index Report 2024.

[7] Eurostat Food Waste and Food Waste Prevention—Estimates. Ec.europa.eu. https://ec.europa.eu/eurostat/statistics-explained/index.php?title=Food_waste_and_food_waste_prevention_-_estimates.

[8] Parfitt J., Barthel M., Macnaughton S. (2010). Food Waste within Food Supply Chains: Quantification and Potential for Change to 2050. Philos. Trans. R. Soc. B Biol. Sci..

[9] Gustavsson J., Cederberg C., Sonesson U., van Otterdijk R., Meyneck A. (2011). Global Food Losses and Food Waste: Extent, Causes and Prevention.

[10] Derqui B., Fernandez V., Fayos T. (2018). Towards More Sustainable Food Systems. Addressing Food Waste at School Canteens. Appetite.

[11] Ministero dell’Ambiente e della Tutela del Territorio e del Mare (2011). Adozione Dei Criteri Minimi Ambientali Da Inserire Nei Bandi Di Gara Della Pubblica Amministrazione per l’acquisto Di Prodotti e Servizi Nei Settori Della Ristorazione Collettiva e Fornitura Di Derrate Alimentari e Serramenti Esterni.

[12] The European Parliament and the Council of the European Union (2014). Directive 2014/24/EU of the European Parliament and of the Council of 26 February 2014 on Public Procurement and Repealing Directive 2004/18/EC.

[13] Favuzzi N., Trerotoli P., Forte M.G., Bartolomeo N., Serio G., Lagravinese D., Vino F. (2020). Evaluation of an Alimentary Education Intervention on School Canteen Waste at a Primary School in Bari, Italy. Int. J. Environ. Res. Public Health.

[14] Cooper K.A., Quested T.E., Lanctuit H., Zimmermann D., Espinoza-Orias N., Roulin A. (2018). Nutrition in the Bin: A Nutritional and Environmental Assessment of Food Wasted in the UK. Front. Nutr..

[15] Biasini B., Donati M., Rosi A., Giopp F., Colić Barić I., Bituh M., Brečić R., Brennan M., Ilić A., Quarrie S. (2024). Nutritional, Environmental and Economic Implications of Children Plate Waste at School: A Comparison between Two Italian Case Studies. Public Health Nutr..

[16] Oostindjer M., Aschemann-Witzel J., Wang Q., Skuland S.E., Egelandsdal B., Amdam G.V., Schjøll A., Pachucki M.C., Rozin P., Stein J. (2017). Are School Meals a Viable and Sustainable Tool to Improve the Healthiness and Sustainability of Children’s Diet and Food Consumption? A Cross-National Comparative Perspective. Crit. Rev. Food Sci. Nutr..

[17] Prado E.L., Dewey K.G. (2014). Nutrition and Brain Development in Early Life. Nutr. Rev..

[18] Birch L., Savage J.S., Ventura A. (2007). Influences on the Development of Children’s Eating Behaviours: From Infancy to Adolescence. Can. J. Diet. Pract. Res..

[19] Ballard T.J., Kepple A.W., Cafiero C. (2013). The Food Insecurity Experience Scale: Development of a Global Standard for Monitoring Hunger Worldwide.

[20] Everitt H., Van Der Werf P., Seabrook J.A., Wray A., Gilliland J.A. (2022). The Quantity and Composition of Household Food Waste during the COVID-19 Pandemic: A Direct Measurement Study in Canada. Socio-Econ. Plan. Sci..

[21] Roustit C., Hamelin A.-M., Grillo F., Martin J., Chauvin P. (2010). Food Insecurity: Could School Food Supplementation Help Break Cycles of Intergenerational Transmission of Social Inequalities?. Pediatrics.

[22] Truman E., Lane D., Elliott C. (2017). Defining Food Literacy: A Scoping Review. Appetite.

[23] Jomori M.M., Proença R.P.D.C., Calvo M.C.M. (2008). Determinantes de Escolha Alimentar. Rev. Nutr..

[24] Marty L., Miguet M., Bournez M., Nicklaus S., Chambaron S., Monnery-Patris S. (2017). Do Hedonic- versus Nutrition-Based Attitudes toward Food Predict Food Choices? A Cross-Sectional Study of 6- to 11-Year-Olds. Int. J. Behav. Nutr. Phys. Act..

[25] Dubé L., Cantin I. (2000). Promoting Health or Promoting Pleasure? A Contingency Approach to the Effect of Informational and Emotional Appeals on Food Liking and Consumption. Appetite.

[26] Brecic R., Gorton M., Cvencek D. (2022). Development of Children’s Implicit and Explicit Attitudes toward Healthy Food: Personal and Environmental Factors. Appetite.

[27] Monnery-Patris S., Marty L., Bayer F., Nicklaus S., Chambaron S. (2016). Explicit and Implicit Tasks for Assessing Hedonic-versus Nutrition-Based Attitudes towards Food in French Children. Appetite.

[28] Van Der Heijden A., Te Molder H., Jager G., Mulder B.C. (2021). Healthy Eating Beliefs and the Meaning of Food in Populations with a Low Socioeconomic Position: A Scoping Review. Appetite.

[29] Maimaran M., Fishbach A. (2014). If It’s Useful and You Know It, Do You Eat? Preschoolers Refrain from Instrumental Food. J. Consum. Res..

[30] Shutts K., Kinzler K.D., DeJesus J.M. (2013). Understanding Infants’ and Children’s Social Learning about Foods: Previous Research and New Prospects. Dev. Psychol..

[31] World Health Organization Regional Office for the Eastern Mediterranean (2019). Strategy on Nutrition for the Eastern Mediterranean Region 2020–2030.

[32] Elnakib S.A., Quick V., Mendez M., Downs S., Wackowski O.A., Robson M.G. (2021). Food Waste in Schools: A Pre-/Post-Test Study Design Examining the Impact of a Food Service Training Intervention to Reduce Food Waste. Int. J. Environ. Res. Public Health.

[33] Liu H., Feng S., Hu X. (2022). Process vs. Outcome: Effects of Food Photo Types in Online Restaurant Reviews on Consumers’ Purchase Intention. Int. J. Hosp. Manag..

[34] Ofei K.T., Holst M., Rasmussen H.H., Mikkelsen B.E. (2014). How Practice Contributes to Trolley Food Waste. A Qualitative Study among Staff Involved in Serving Meals to Hospital Patients. Appetite.

[35] LimeSurvey GmbH (2026). LimeSurvey: An Open Source Survey Tool.

[36] Buzby J.C., Guthrie J.F. (2002). Plate Waste in School Nutrition Programs: Final Report to Congress.

[37] Engström R., Carlsson-Kanyama A. (2004). Food Losses in Food Service Institutions Examples from Sweden. Food Policy.

[38] Eriksson M., Persson Osowski C., Malefors C., Björkman J., Eriksson E. (2017). Quantification of Food Waste in Public Catering Services—A Case Study from a Swedish Municipality. Waste Manag..

[39] Antón-Peset A., Fernandez-Zamudio M.-A., Pina T. (2021). Promoting Food Waste Reduction at Primary Schools. A Case Study. Sustainability.

[40] Ministerio de Agricultura, Alimentación y Medio Ambiente (2016). Estudio piloto para la medición y reducción del desperdicio de alimentos en comedores escolares. Diseño de una Auditoría de Autoevaluación.

[41] Boschini M., Falasconi L., Giordano C., Alboni F. (2018). Food Waste in School Canteens: A Reference Methodology for Large-Scale Studies. J. Clean. Prod..

[42] Hunsberger M., McGinnis P., Smith J., Beamer B.A., O’Malley J. (2014). Elementary School Children’s Recess Schedule and Dietary Intake at Lunch: A Community-Based Participatory Research Partnership Pilot Study. BMC Public Health.

[43] Serra-Majem L., Ribas L., Ngo J., Ortega R.M., García A., Pérez-Rodrigo C., Aranceta J. (2004). Food, Youth and the Mediterranean Diet in Spain. Development of KIDMED, Mediterranean Diet Quality Index in Children and Adolescents. Public Health Nutr..

[44] Archero F., Ricotti R., Solito A., Carrera D., Civello F., Di Bella R., Bellone S., Prodam F. (2018). Adherence to the Mediterranean Diet among School Children and Adolescents Living in Northern Italy and Unhealthy Food Behaviors Associated to Overweight. Nutrients.

[45] Mariscal-Arcas M., Rivas A., Velasco J., Ortega M., Caballero A.M., Olea-Serrano F. (2009). Evaluation of the Mediterranean Diet Quality Index (KIDMED) in Children and Adolescents in Southern Spain. Public Health Nutr..

[46] López-Gajardo M.A., Leo F.M., Sánchez-Miguel P.A., López-Gajardo D., Soulas C., Tapia-Serrano M.A. (2022). KIDMED 2.0, An Update of the KIDMED Questionnaire: Evaluation of the Psychometric Properties in Youth. Front. Nutr..

[47] Štefan L., Prosoli R., Juranko D., Čule M., Milinović I., Novak D., Sporiš G. (2017). The Reliability of the Mediterranean Diet Quality Index (KIDMED) Questionnaire. Nutrients.

[48] Sindhu B.S., Shechtman O., Tuckey L. (2011). Validity, Reliability, and Responsiveness of a Digital Version of the Visual Analog Scale. J. Hand Ther..

[49] De Wildt K.K., Meijers M.H.C. (2023). Time Spent on Separating Waste Is Never Wasted: Fostering People’s Recycling Behavior through the Use of a Mobile Application. Comput. Hum. Behav..

[50] Bradley M.M., Lang P.J. (1994). Measuring Emotion: The Self-Assessment Manikin and the Semantic Differential. J. Behav. Ther. Exp. Psychiatry.

[51] Sorokowska A., Marczak M., Misiak M., Stefańczyk M.M., Sorokowski P. (2020). Children Older than Five Years Do Not Approve of Wasting Food: An Experimental Study on Attitudes towards Food Wasting Behavior in Children and Adults. J. Environ. Psychol..

[52] Bartosova M., Svetlak M., Kukletova M., Borilova Linhartova P., Dusek L., Izakovicova Holla L. (2019). Emotional Stimuli Candidates for Behavioural Intervention in the Prevention of Early Childhood Caries: A Pilot Study. BMC Oral Health.

[53] DeJesus J.M., Gelman S.A., Lumeng J.C. (2020). Children’s Implicit Food Cognition: Developing a Food Implicit Association Test. Cogn. Dev..

[54] Weisberg H.F., Miller A.H. (1979). Evaluation of the Feeling Thermometer: A Report to the ANES Board Based on Data from the 1979 Pilot Study Survey.

[55] Van Der Heijden A., Te Molder H., De Graaf C., Jager G. (2020). Healthy Is (Not) Tasty? Implicit and Explicit Associations between Food Healthiness and Tastiness in Primary School-Aged Children and Parents with a Lower Socioeconomic Position. Food Qual. Prefer..

[56] Foroni F., Pergola G., Argiris G., Rumiati R.I. (2013). The FoodCast Research Image Database (FRIDa). Front. Hum. Neurosci..

[57] Blechert J., Meule A., Busch N.A., Ohla K. (2014). Food-Pics: An Image Database for Experimental Research on Eating and Appetite. Front. Psychol..

[58] Villalonga-Olives E., Kiese-Himmel C., Witte C., Almansa J., Dusilova I., Hacker K., Von Steinbuechel N. (2015). Self-Reported Health-Related Quality of Life in Kindergarten Children: Psychometric Properties of the Kiddy-KINDL. Public Health.

[59] Ravens-Sieberer U., Bullinger M. (1998). Assessing Health-Related Quality of Life in Chronically Ill Children with the German KINDL: First Psychometric and Content Analytical Results. Qual. Life Res..

[60] Italian: Questionnaire Versions. https://www.kindl.org/contacts/italian/.

[61] Altavilla C., Caballero-Pérez P. (2019). An Update of the KIDMED Questionnaire, a Mediterranean Diet Quality Index in Children and Adolescents. Public Health Nutr..

[62] R core Team (2026). R: A Language and Environment for Statistical Computing.

[63] Jones M., Dailami N., Weitkamp E., Salmon D., Kimberlee R., Morley A., Orme J. (2012). Food Sustainability Education as a Route to Healthier Eating: Evaluation of a Multi-Component School Programme in English Primary Schools. Health Educ. Res..

[64] Carlsson L., Williams P.L. (2008). New Approaches to the Health Promoting School: Participation in Sustainable Food Systems. J. Hunger Environ. Nutr..

[65] Dos Santos E.B., Da Costa Maynard D., Zandonadi R.P., Raposo A., Botelho R.B.A. (2022). Sustainability Recommendations and Practices in School Feeding: A Systematic Review. Foods.

[66] Wardle J., Cooke L.J., Gibson E.L., Sapochnik M., Sheiham A., Lawson M. (2003). Increasing Children’s Acceptance of Vegetables; a Randomized Trial of Parent-Led Exposure. Appetite.

[67] Nekitsing C., Hetherington M.M., Blundell-Birtill P. (2018). Developing Healthy Food Preferences in Preschool Children Through Taste Exposure, Sensory Learning, and Nutrition Education. Curr. Obes. Rep..

[68] Cooke L. (2007). The Importance of Exposure for Healthy Eating in Childhood: A Review. J. Hum. Nutr. Diet..

[69] Mennella J.A., Nicklaus S., Jagolino A.L., Yourshaw L.M. (2008). Variety Is the Spice of Life: Strategies for Promoting Fruit and Vegetable Acceptance during Infancy. Physiol. Behav..

[70] Fildes A., Van Jaarsveld C.H.M., Wardle J., Cooke L. (2014). Parent-Administered Exposure to Increase Children’s Vegetable Acceptance: A Randomized Controlled Trial. J. Acad. Nutr. Diet..

[71] Cooke L.J., Wardle J. (2005). Age and Gender Differences in Children’s Food Preferences. Br. J. Nutr..

[72] Kimura S., Endo Y., Minamimae K., Kanzaki S., Hanaki K. (2014). Gender Differences in Childhood Food Preference: Evaluation Using a Subjective Picture Choice Method. Pediatr. Int..

