# Supplementary material for: An Intervention to Promote Sustainable and Inclusive Eating Behaviors in Primary and Preschools in Italy: A Pilot Study of the SoS-4-SoUPS Project
Source: Nutrients. 2026 Nov 7;18(21):3700. doi: 10.3390/nu18213700 (PMC13765376; doi:10.3390/nu18213700)
Supplement: Supplementary file 1 [file nutrients-18-03700-s001.zip › Supplmentary Article_Vedani_Habib.pdf]

Supplementary materials

Table S3. Baseline characteristics according to follow-up questionnaire participation.

Baseline characteristic	T1 respondents (n = 48)	T1 non-respondents (n = 45)	Mean difference [95% CI]	Raw p
**Child age (years)**	7.09 (2.19)	7.44 (2.60)	-0.36 [-1.43, 0.71]	0.505
**KIDMED**	5.62 (2.11)	5.07 (2.49)	0.56 [-0.40, 1.51]	0.248
**KINDL**	79.31 (11.90)	75.25 (11.96)	4.05 [-1.35, 9.45]	0.139
**Parental VAS relevance**	8.95 (1.95)	8.37 (2.52)	0.59 [-0.35, 1.52]	0.216
**Parental VAS arousal**	7.76 (2.55)	7.79 (2.47)	-0.02 [-1.06, 1.01]	0.963
**Parental VAS dominance**	6.25 (2.64)	7.32 (2.12)	-1.07 [-2.05, -0.09]	0.033
**SAM valence**	2.00 (1.15)	2.44 (1.40)	-0.44 [-1.00, 0.12]	0.122
**SAM arousal**	5.50 (2.55)	5.01 (2.03)	0.49 [-0.54, 1.52]	0.348
**SAM dominance**	4.83 (2.63)	5.51 (2.25)	-0.68 [-1.77, 0.41]	0.215
**Food images: HC**	7.84 (1.93)	7.54 (1.54)	0.30 [-0.48, 1.08]	0.443
**Food images: LC**	5.38 (2.73)	5.07 (2.48)	0.31 [-0.84, 1.47]	0.590
**Perceived inclusivity**	6.06 (2.92)	5.53 (3.13)	0.53 [-0.72, 1.78]	0.402
**Perceived involvement**	2.71 (2.97)	4.44 (3.44)	-1.74 [-3.07, -0.41]	0.011
**Child sex: F**	22/48	23/45		0.611
**Child sex: M**	26/48	22/45		

Continuous values: mean (SD)

Mean difference = respondents minus non-respondents. Confidence intervals are unadjusted.

Welch t-tests for continuous measures; Pearson chi-square tests for sex and school.

Table S3. Baseline characteristics of participants with follow-up questionnaire data and participants assessed at baseline only.

Residual and Sensitivity Analysis:

Residuals of the linear mixed models were inspected using Q-Q plots, residuals-vs-fitted plots, skewness, kurtosis and Shapiro-Wilk tests (Supplementary Figures Model 1– Model 9). Residual skewness/kurtosis were −0.51/6.81 for parents' VAS arousal, −1.63/13.79 for relevance, −0.17/4.21 for dominance, −0.34/2.42 for perceived inclusivity, 0.29/2.16 for perceived involvement, −0.26/4.27 for children's SAM arousal, 1.15/6.35 for valence, −0.27/3.38 for dominance, and −0.48/2.88 for the Feeling Thermometer. Shapiro-Wilk tests were significant for all models (W between 0.74 and 0.99, all $p < .01$; e.g., relevance: $W = 0.739$, arousal: $W = 0.877$). VAS scale on relevance showed the strongest departure from normality, owing to a marked ceiling effect (64% of responses equal to 10).

As a sensitivity analysis, the models on parents' VAS and children's SAM and Feeling Thermometer ratings were refitted as cumulative-link mixed models. Effects are expressed as odds ratios (OR) with 95% confidence intervals, where OR > 1 indicates higher ratings. For parents' VAS, results were consistent with the linear models for dominance (OR = 1.16, 95% CI [0.82, 1.63], $p = .405$) and for perceived inclusivity and involvement, which were higher at T1 (inclusivity: OR = 2.31, 95% CI [1.25, 4.26], $p = .007$; involvement: OR = 4.13, 95% CI [2.27, 7.54], $p < .001$). For arousal, ratings tended to be higher at T1, in line with the linear model, whose residuals showed heavy tails (kurtosis = 6.81), but the effect was at the margin of conventional significance (OR = 1.47, 95% CI [0.99, 2.19], $p = .055$). For relevance, where residuals were markedly skewed and heavy-tailed, the linear model detected no change in mean ratings, whereas the cumulative-link model indicated a small shift of the distribution towards lower ratings at T1 (OR = 0.49, 95% CI [0.31, 0.77], $p = .002$); among the 48 parents with data at both time points, the proportion of maximum ratings decreased from 70% to 58.3%, while the mean appeared stable (8.95 vs. 8.87). For children's SAM, the main results of the linear models were confirmed. Arousal was higher at T1 (OR = 1.80, 95% CI [1.22, 2.65], $p = .003$) and higher in primary school children than in preschoolers (OR = 8.99, 95% CI [1.76, 46.02], $p = .008$), with no Time × Gender interaction (likelihood-ratio test: $\chi^2(1) = 0.07$, $p = .787$). The Time × Gender interaction was confirmed for valence ($\chi^2(1) = 8.46$, $p = .004$), with an increase in females (OR = 3.46, 95% CI [1.73, 6.93], $p < .001$) and no change in males (OR = 0.92, 95% CI [0.50, 1.68], $p = .775$), and for dominance ($\chi^2(1) = 8.12$, $p = .004$), with opposite directions of change in females (OR = 1.88, 95% CI [1.08, 3.28], $p = .025$) and males (OR = 0.62, 95% CI [0.36, 1.05], $p = .077$). After Bonferroni correction neither dominance change was significant

(p = .051 and p = .153), so the significant decrease in males found in the linear model should be interpreted with caution; the effect of School Grade on dominance was reduced to a trend (OR = 4.81, 95% CI [0.90, 25.73], p = .066). For the Feeling Thermometer, Time had no effect (OR = 0.90, 95% CI [0.58, 1.41], p = .647) and did not interact with Food Category or Gender (all p > .58), and the Food Category × Gender interaction was confirmed (OR = 0.36, 95% CI [0.22, 0.58], p < .001): indicating that the difference between low- and high-calorie food was larger in males than in females. At T0, low-calorie food was rated lower than high-calorie food in females (OR = 0.39, 95% CI [0.20, 0.76], p = .006), and no gender difference emerged for high-calorie food (OR = 1.29, 95% CI [0.81, 2.05], p = .292). The only effect that differed was that of Age, which was not significant in the linear model (p = .095) but reached significance in the cumulative-link model (OR = 0.88, 95% CI [0.78, 0.99], p = .032).

Figure S1 : VAS arousal

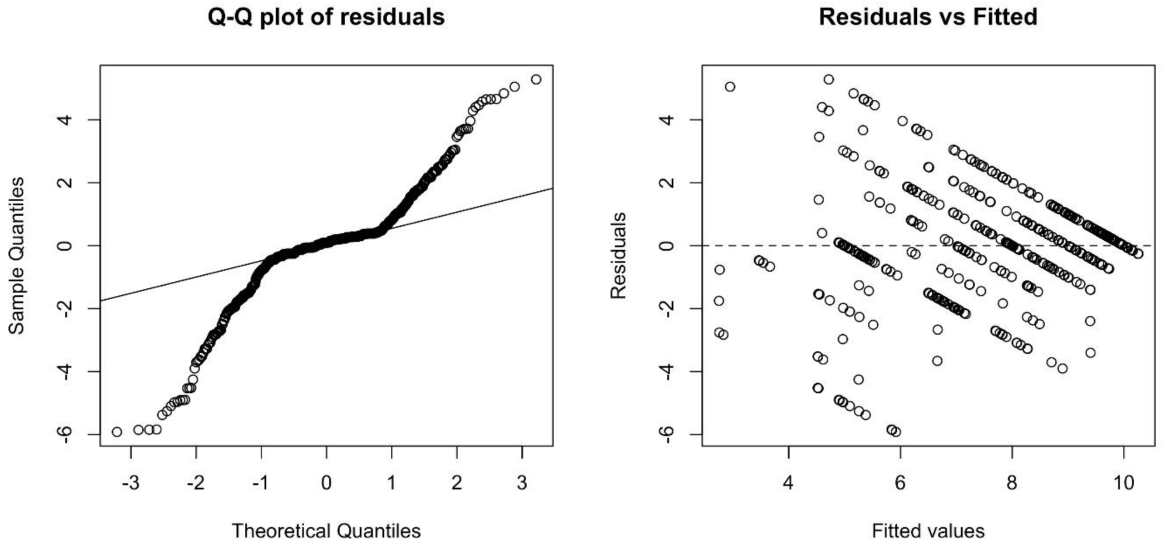

Figure S1: Model diagnostic plots: QQ-plot of VAS arousal residuals to assess normality, and residuals versus fitted values plot to evaluate homoscedasticity and model adequacy.

Figure S2 : VAS valence

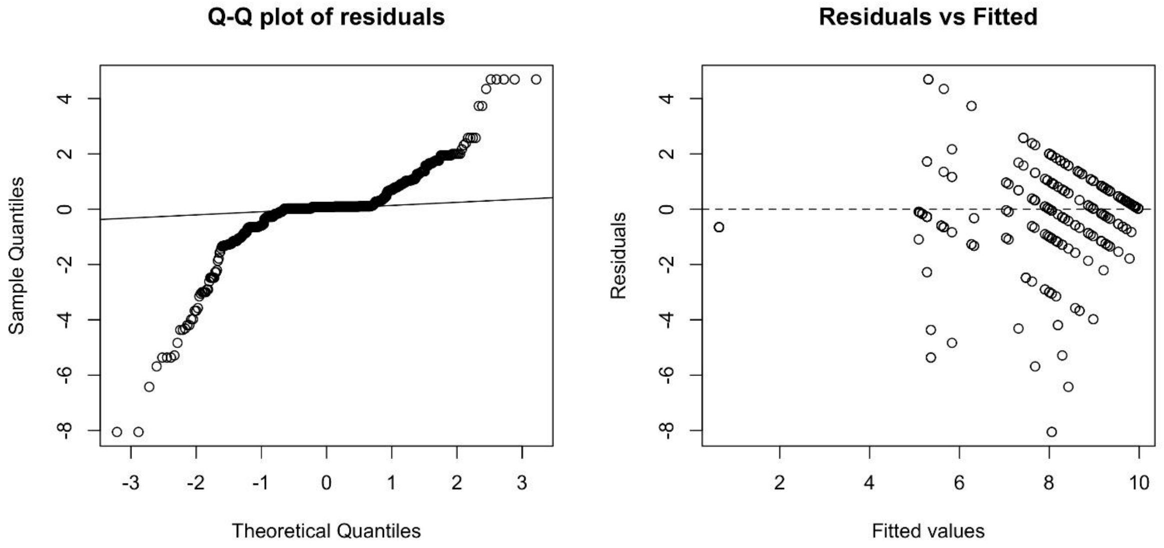

Figure S2: Model diagnostic plots: QQ-plot of VAS valence residuals to assess normality, and residuals versus fitted values plot to evaluate homoscedasticity and model adequacy.

Figure S3: VAS dominance

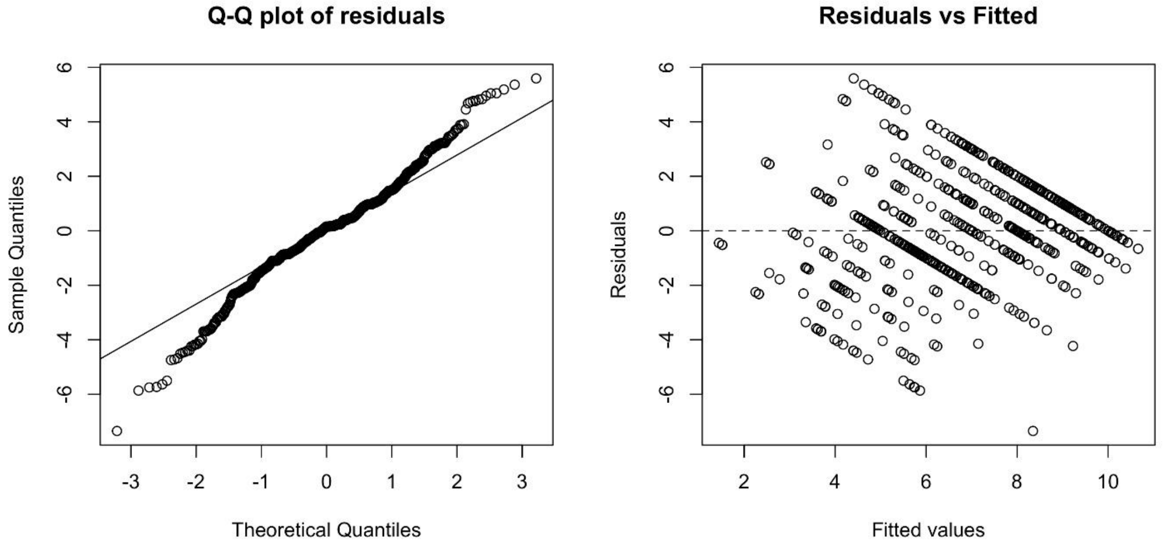

Figure S3: Model diagnostic plots: QQ-plot of VAS dominance residuals to assess normality, and residuals versus fitted values plot to evaluate homoscedasticity and model adequacy.

Figure S4: SAM arousal

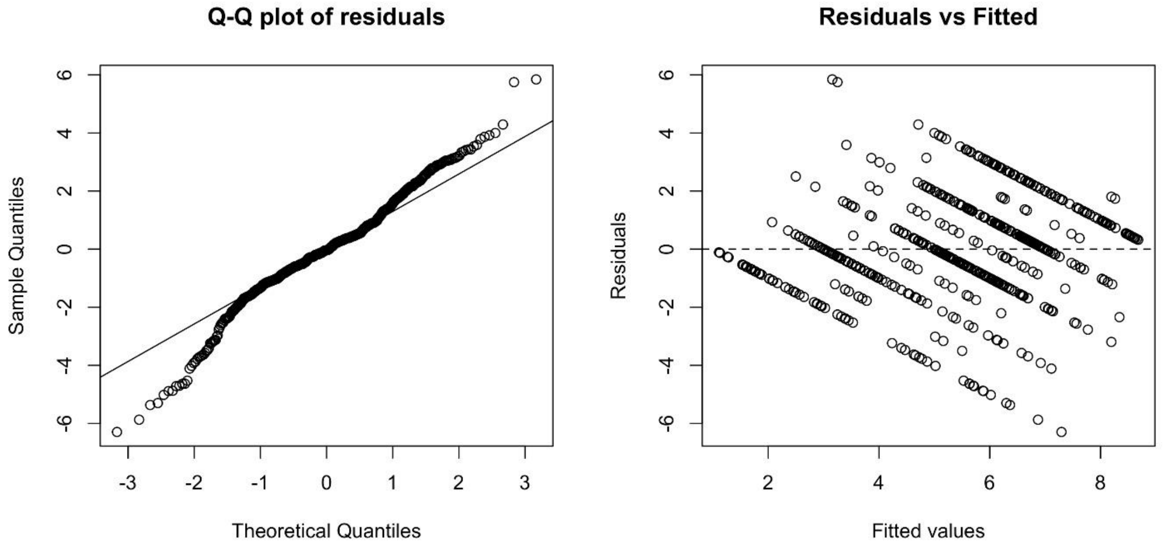

Figure S4: Model diagnostic plots: QQ-plot of SAM arousal residuals to assess normality, and residuals versus fitted values plot to evaluate homoscedasticity and model adequacy.

Figure S5: SAM Valence

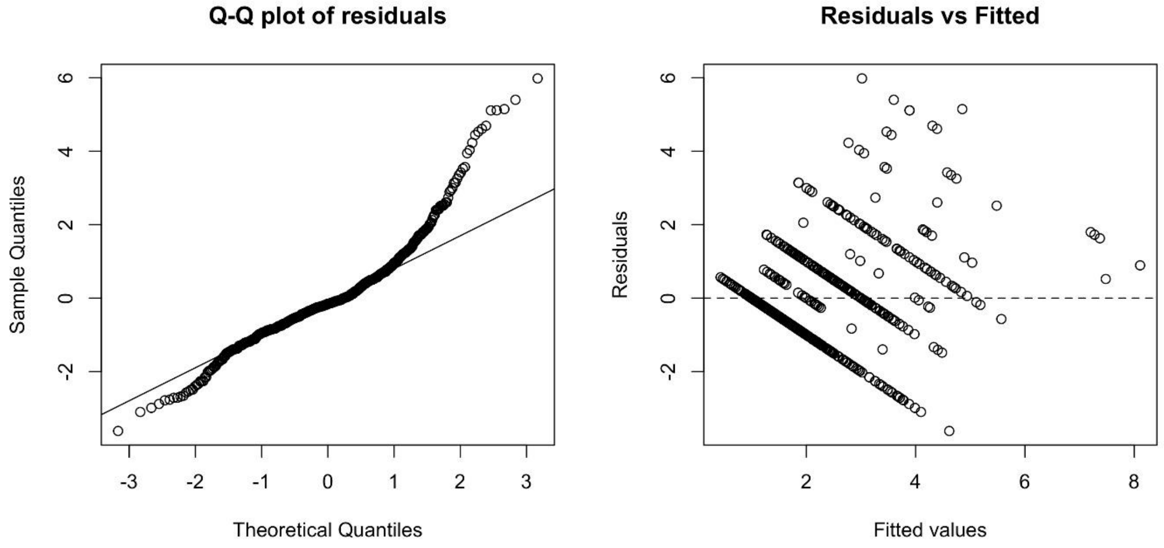

Figure S5: Model diagnostic plots: QQ-plot of SAM valence residuals to assess normality, and residuals versus fitted values plot to evaluate homoscedasticity and model adequacy.

Figure S6: SAM dominance

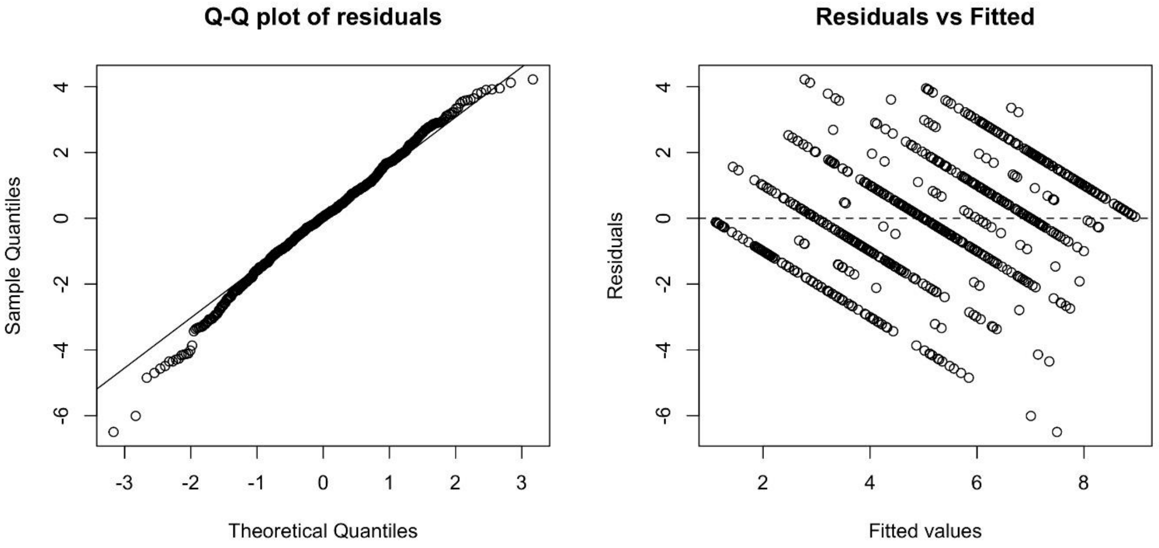

Figure S6: Model diagnostic plots: QQ-plot of SAM dominance residuals to assess normality, and residuals versus fitted values plot to evaluate homoscedasticity and model adequacy.

Figure S7: VAS inclusivity

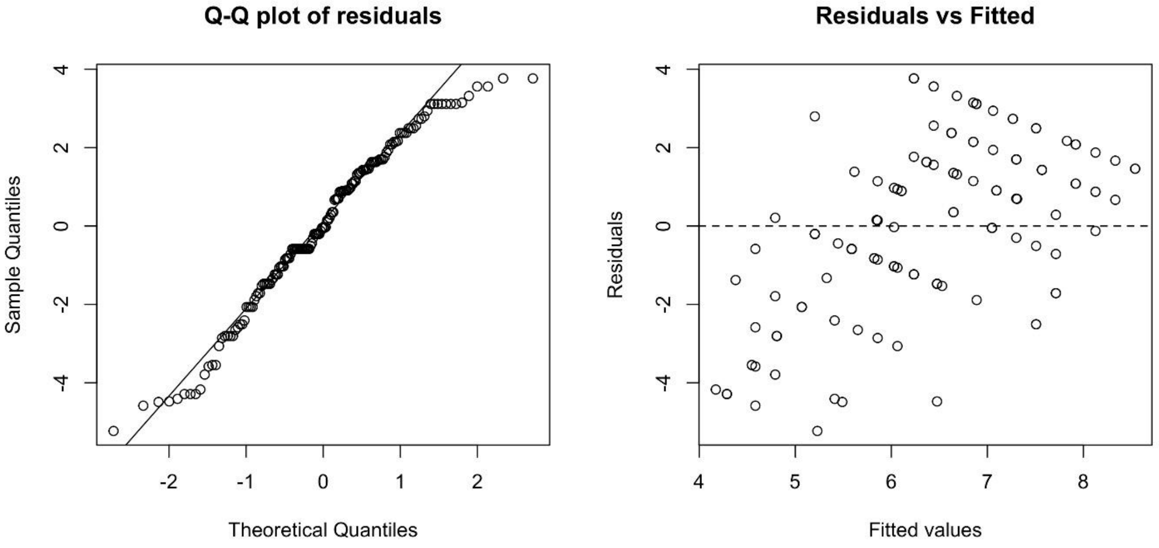

Figure S7: Model diagnostic plots: QQ-plot of VAS inclusivity residuals to assess normality, and residuals versus fitted values plot to evaluate homoscedasticity and model adequacy.

Figure S8: VAS involvement

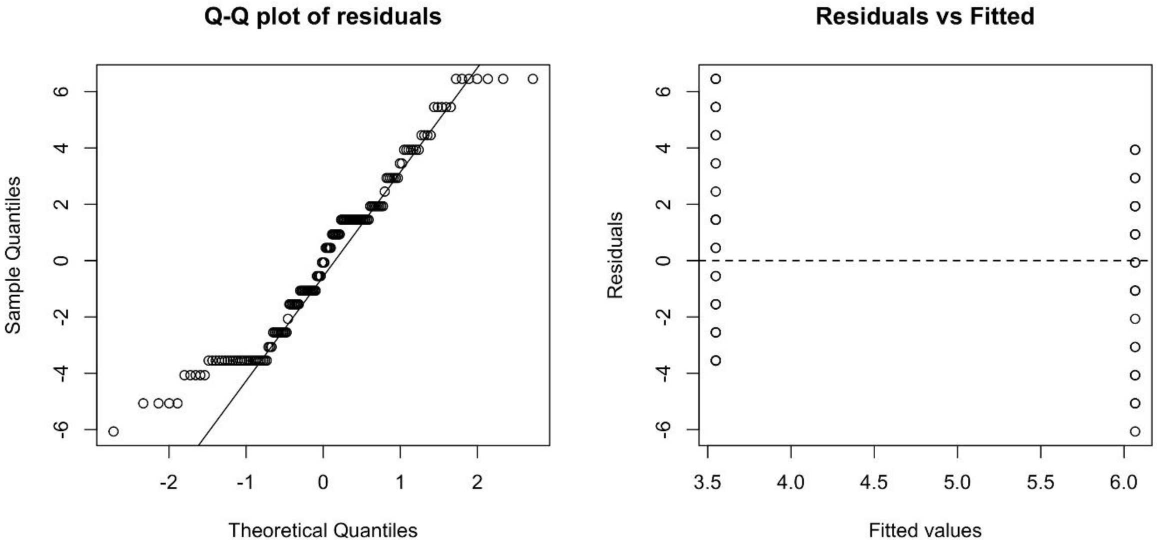

Figure S8: Model diagnostic plots: QQ-plot of VAS involvement residuals to assess normality, and residuals versus fitted values plot to evaluate homoscedasticity and model adequacy.

Figure S9: Feeling Thermometer

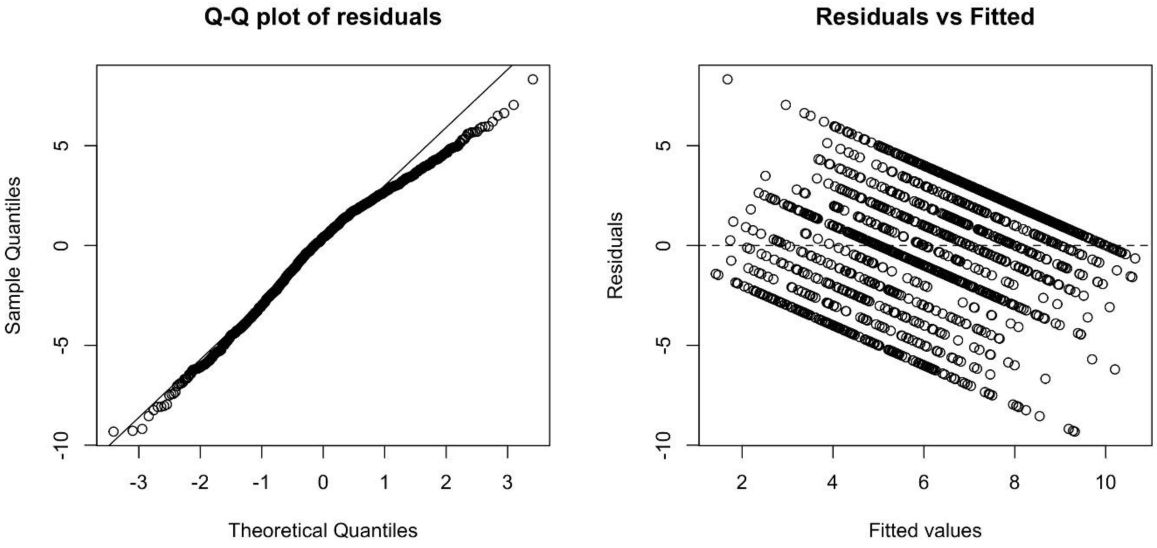

Figure S9: Model diagnostic plots: QQ-plot of Feeling Thermometer residuals to assess normality, and residuals versus fitted values plot to evaluate homoscedasticity and model adequacy.
